# Triboelectric Nanogenerators for Future Space Missions

**DOI:** 10.1007/s40820-025-01944-5

**Published:** 2026-01-04

**Authors:** Rayyan Ali Shaukat, Muhammad Muqeet Rehman, Maryam Khan, Rui Chang, Carlo Saverio Iorio, Yarjan Abdul Samad, Yijun Shi

**Affiliations:** 1 Division of Machine Elements, Lulea University of Technology, 97187 Lulea, Sweden; 2 Department of Electronic Engineering, Faculty of Applied Energy System, Jeju National University, Jeju City, 63243 Republic of Korea; 3 Aerospace Research and Innovation Center (ARIC), Khalifa University of Science and Technology, 127788 Abu Dhabi, United Arab Emirates; 4 Centre for Research and Engineering in Space Technologies, University of Brussels, Brussels , Belgium; 5 Department of Aerospace Engineering, Khalifa University of Science & Technology, 127788 Abu Dhabi, United Arab Emirates; 6 Engineering Department, Cambridge Graphene Centre, University of Cambridge, Cambridge, UK; 7 Faculty of Electrical Engineering, Ghulam Ishaq Khan Institute of Engineering Sciences and Technology, 23640 Topi, Pakistan

**Keywords:** Triboelectric nanogenerators (TENGs), Space missions, Sustainable energy harvesting, Harsh space conditions, Self-powered sensors, Satellite missions

## Abstract

Space exploration is significant for scientific innovation, resource utilization, and planetary security. Space exploration involves several systems including satellites, space suits, communication systems, and robotics, which have to function under harsh space conditions such as extreme temperatures (− 270 to 1650 °C), microgravity (10⁻⁶ g), unhealthy humidity (< 20% RH or > 60% RH), high atmospheric pressure (~ 1450 psi), and radiation (4000–5000 mSv). Conventional energy-harvesting technologies (solar cells, fuel cells, and nuclear energy), that are normally used to power these space systems have certain limitations (e.g., sunlight dependence, weight, degradation, big size, high cost, low capacity, radioactivity, complexity, and low efficiency). The constraints in conventional energy resources have made it imperative to look for non-conventional yet efficient alternatives. A great potential for enhancing efficiency, sustainability, and mission duration in space exploration can be offered by integrating triboelectric nanogenerators (TENGs) with existing energy sources. Recently, the potential of TENG including energy harvesting (from vibrations/movements in satellites and spacecraft), self-powered sensing, and microgravity, for multiple applications in different space missions has been discussed. This review comprehensively covers the use of TENGs for various space applications, such as planetary exploration missions (Mars environment monitoring), manned space equipment, In-orbit robotic operations /collision monitoring, spacecraft's design and structural health monitoring, Aeronautical systems, and conventional energy harvesting (solar and nuclear). This review also discusses the use of self-powered TENG sensors for deep space object perception. At the same time, this review compares TENGs with conventional energy harvesting technologies for space systems. Lastly, this review talks about energy harvesting in satellites, TENG-based satellite communication systems, and future practical implementation challenges (with possible solutions).

## Highlights

- This review paper highlights the comprehensive evaluation of triboelectric nanogenerators (TENGs) for various space environments.
- This review paper discusses the multifunctional role of TENGs beyond energy harnessing.
- This review demonstrates the future trends (possible roadmap) of utilization of TENGs in space exploration.

## Graphical abstract

**Figure:**
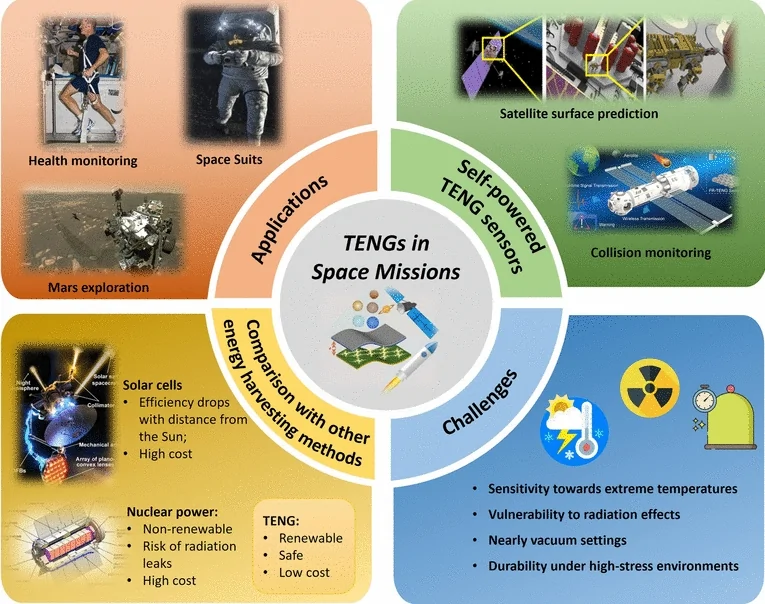

## Introduction

Space exploration and satellite systems depend critically on advanced energy harvesting and autonomous sensing technologies to endure and operate effectively in the extreme conditions of outer space. Temperature fluctuations during space missions could be dramatically extreme: in shadowed regions, temperatures can depreciate to approximately − 270 °C (− 454 °F), while the spacecraft can face an extremely high temperature of ~ 1650 °C as rentering into earth’s atmosphere. Space itself is an almost perfect vacuum with pressures averaging between 10⁻^12^ and 10⁻^15^ torr, compared to Earth's atmospheric pressure of 760 torr [1]. Astronauts in low Earth orbit (LEO) are exposed to cosmic radiation levels of ~ 0.1–0.2 Sieverts (Sv) annually (higher than Earth's average of 0.01 Sv). During solar flares, radiation levels can spike dramatically, reaching several hundred times normal rates. In LEO, while gravity is approximately 90% of Earth's, astronauts experience microgravity conditions (~ 10⁻⁶ g). The lunar surface further complicates space exploration due to its dust, composed of highly abrasive particles ranging from microns to millimeters in size [2, 3]. Moreover, the Van Allen radiation belts present a significant hazard to both satellites and astronauts, with inner belt (700–12,000 km altitude) radiation levels exceeding 1–5 mGy/day while 0.1–1 mGy/day in the outer belt (13,000–60,000 km altitude). Such extreme conditions render severe challenges to conventional energy systems, making them unsuitable for long space missions and hence, necessitating innovative solutions tailored to the unique demands of space environments.

Space systems, like satellites and spacecraft, usually rely on energy sources such as solar panels, radioisotope thermoelectric generators (RTGs), and fuel cells [4–8]. Solar panels are used because they convert abundantly available sunlight directly into electricity through photovoltaic cells, thus providing a constant and sustainable power supply (perfect for missions in orbit) [9]. Rechargeable batteries store energy harvested from solar panels, ensuring a continuous power supply for onboard systems even during periods of darkness (when a satellite passes through Earth's shadow). RTGs are another vital energy source utilized in space missions, particularly those with limited sunlight exposure. RTGs convert heat produced by the radioactive decay of isotopes into electricity, making them ideal for long-duration space missions, such as those of Mars rovers and Voyager probes (multiple years), where solar energy may be insufficient [5–10].

The above-mentioned conventional energy sources (solar cells, fuel cells, and RTGs) for space systems have their limitations that are slowing down the advancement of space exploration [11]. Limitations of using solar panels include their huge reliance on sunlight that could not work well in the low or no light areas of Earth [9]. Therefore, heavy batteries must be used to store the energy generated by solar cells. These batteries could negatively impact the spacecraft’s operational time during extended periods without sunlight (when it is in Earth's shadow or during eclipses) [12]. Moreover, high-capacity batteries can increase the overall weight and fuel requirements of space systems. This might lead to an increased launch cost and complex designed requirements. The efficiency of this conventional energy system will also be compromised with passing time due to repeated charge cycles and exposure to harsh space conditions, resulting in reduced efficiency and a shorter lifespan. RTGs, while reliable, also come with significant drawbacks during space applications. The use of radioactive isotopes raises safety concerns, particularly during launch, as any malfunction could lead to the release of hazardous materials (that could be lethal). Additionally, their operation requires stringent handling protocols to ensure the safety of both equipment and personnel. These challenges underscore the need for robust containment systems and thorough risk management strategies in missions utilizing RTGs [9]. RTGs typically generate power that might not be adequate for energy-intensive systems. Furthermore, the development and integration of RTGs could also increase the cost of space missions, while the specialized design required for RTGs introduces complexity to spacecraft engineering. RTGs produce immense heat that requires additional thermal control systems, and the decay of isotopes necessitates meticulous mission planning. These drawbacks underscore the necessity for innovative and non-conventional energy storage options for space systems.

Triboelectric nanogenerators (TENGs) could offer a possible solution to the challenges faced by conventional energy sources in space technology. TENGs could effectively transform mechanical energy from movements and environmental fluctuations (generated by spacecraft activities and micrometeoroid collisions) into electrical energy [13]. TENGs can reduce reliance on conventional energy storage in scenarios when solar panels are less efficient, serving as handy supplementary power sources during eclipses or low-light situations. Their lightweight and compact design allows easy integration into space systems without adding significant weight [11]. The compact and lightweight nature of TENGs makes it feasible to incorporate them into spacecraft without being burdened by large and heavy batteries. The robust structure of TENG devices allows them to withstand harsh space conditions [14–16], thus enhancing their reliability and reducing maintenance requirements [17, 18]. Through the development of renewable energy sources, TENG technology aims at decreasing both energy generation and storage costs, which makes sustainable energy more accessible. Research carried out both in microgravity and at the International Space Station (ISS) proves that this concept is feasible for space missions, which makes it a big step forward for future space exploration.

**Fig. 1:**
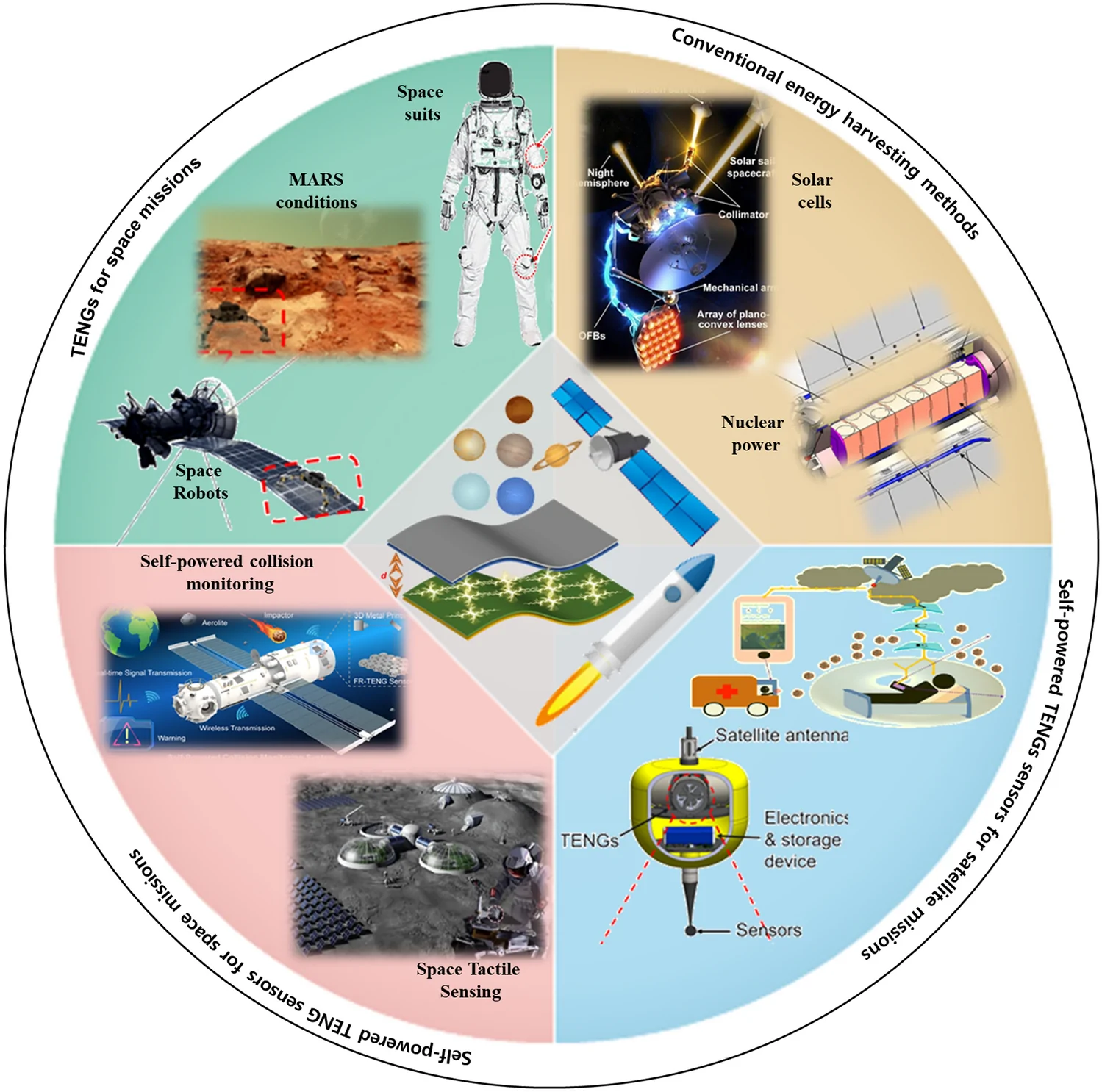
Schematic overview of the conventional energy-generating technologies, TENGs for space missions, self-powered TENG sensors for space missions, and self-powered triboelectric sensors for satellite operations

In spite of its importance and the huge impact that it could have on future space missions, only a little work has been done on discussing the role of TENGs in space systems. This review paper, for the first time, offers an analysis of TENGs by discussing their use in space systems like satellites and space missions, as shown in Fig. 1. Focus of this review includes but is not limited to material selection for TENGs used in space missions, conventional energy sources (nuclear and solar) for space missions, and integration of TENGs in planetary exploration missions, spacecraft’s health monitoring, manned space equipment, aeronautical systems, and in-orbit operations/mission management. This review paper also focuses on self-powered sensors based on TENGs to support space exploration, TENG’s application in satellite communication, and future prospects of this non-conventional technology. We believe that this review will encourage several young researchers globally to participate in this promising field and contribute to the question of how TENGs are going to make future space exploration missions more successful for the betterment of the human race.

### Basic Principles and Working Modes of the TENGs

TENGs have emerged as an accelerating technology to harness mechanical energy from the environment to convert it into electrical power through the combination of triboelectrification and electrostatic induction [19]. TENGs offered a promising route toward sustainable and self-powered systems across various applications, including wearable electronics [20, 21], Internet of Things (IoT) devices [22], biomedical devices [23], wind energy utilization [24], and energy harvesting in harsh environments [23]. The basic idea behind TENGs was in its triboelectric effect, which involves the generation of static electricity through the contact/separation of two materials with different electro-negativities [25, 26]. The contact between two different materials results in the transfer of charges between them [27]. When triboelectric materials start to separate from each other, they produce a potential difference and an electric field that drives electrons through a circuit. These devices typically possess two main parts such as triboelectric (positive/negative) layers and electrodes. The triboelectric layers consist of materials with distinct electron affinities (metals, polymers, and ceramics), which show triboelectrification upon mechanical stress or external force. The electrodes facilitate the collection of produced charges to an external circuit or external storage device [28].

**Fig. 2:**
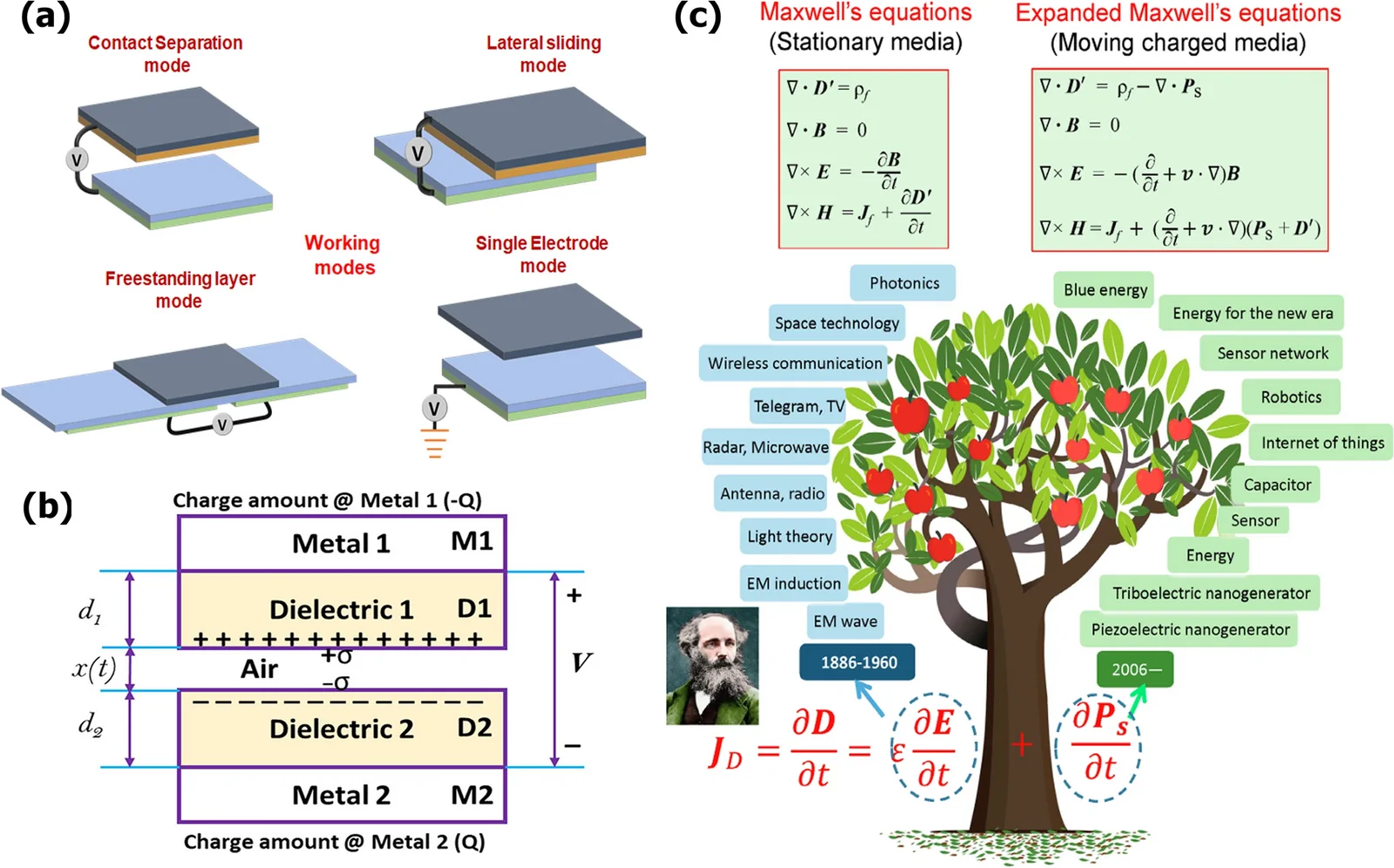
**a** Fundamentals of the working modes of TENGs; **b** schematic of the working mechanism of contact separation TENG’s model (V-Q-x relationship), and **c** schematics of comparison of the fundamentals Maxwell equations and expanded Maxwell equations proposed by Prof. Wang for the foundation of the TENGs, reproduced with permission from American Chemical Society, Copyright@2023 [59]

Four TENG working modes, including single-electrode, freestanding, contact-separation, sliding, and hybrid modes, have been demonstrated to work well [23–25]. In the single-electrode mode, only one electrode is used for charge collection, while the other electrode remains grounded [32], as shown in Fig. 2a. The advantage of this mode is simple, but its limitation is lower output power compared to other modes [32]. In some recent works related to development of tactile sensors for smart extravehicular activities of astronauts, single-electrode mode has been utilized owing to the compatibility with textile substrates, make it suitable for body integration, wearable and stretchable applications. Moreover, this mode was selected in manned space equipment’s owing to simple fabrication design to and has high sensitivity to low-frequency periodic motions. Additionally, in the freestanding mode, both triboelectric layers are hung in a vacuum or air, allowing for independent movement and deformation during mechanical stress [33], as shown in Fig. 2a. This mode enhances the device's sensitivity to external stimuli and reduces parasitic losses from external damping. On the other hand, the contact-separation mode relies on continuous contact and separation between the triboelectric layers to induce triboelectrification and electrostatic induction [34], as demonstrated in Fig. 2a. In several key studies related to Martian conditions, the contact separation mode is widely used configuration owing to its simplicity and suitability for harnessing the energy under harsh Martian conditions. The sliding mode involves continuous sliding or rubbing motion between the triboelectric layers, resulting in sustained triboelectric charging and electrical output as depicted in Fig. 2a [35]. This mode is commonly used in wearable energy harvesters and self-powered sensors. The sliding mode is most suitable for scenarios like crawling robots or robotic bio-paws, where continuous surface interaction and multi-dimensional sensing are needed. Additionally, hybrid modes combining multiple operating principles have been proposed to enhance the performance of TENGs under different environmental conditions and mechanical stimuli [36]. By investigating the different modes of operation of TENGs, researchers/engineers can realize the full potential of this novel technology for energy harvesting and self-powered sensing in diverse real-world applications. Hence, each working modes has its own advantages for space missions/applications depends upon the mechanical stimulus and environmental conditions. Thus, TENG mode selection should be guided by specific application scenarios.

In addition, TENG devices normally operates on four fundamental working mechanisms such as contact-separation, single electrode mode, freestanding and lateral standing modes. However, recent developments have enhanced their functionality for more advanced applications such as space explorations. The most notable advancement is liquid–metal-based TENGs, utilizing liquid metals as flexible conductive electrodes. This liquid metal based TENGs can be suitable for conformal devices on spacecraft. Another advancement is the demonstration of flexible and stretchable TENGs, utilizing the elastomeric substrates (e.g., PDMS, Ecoflex). These innovative TENGs can reliably operates under dynamic movement, and can be integrated with space suits and space gloves where mechanical flexibility is necessary. Hence, these emerging mechanisms significantly enhances the reliability and compatibility of TENG devices beyond the conventional energy harvesters capable of adaptive working in the harsh environments linked with aerospace and deep space missions.

The theoretical working mechanism of the TENG can be explained by Gauss's Law. The charge voltage motion (V-Q-x) can be explained as a time correlation [29]. As shown in Fig. 2b, the triboelectric materials have the thickness of D_1_ and D_2_ with their relative permittivity of ɛ_1_ and ɛ_2_, respectively. When an external constant force is applied to the device, the separation distance (x) changes according to the time. Both the materials with thicknesses of D_1_ and D_2_ come close to each other, and opposite static charges are produced with a charge density of (ρ) on the inner side of the surface. The electric field strength at each dielectric surface is given by:

1 $${\text{Inside Dielectric 1}}:E_{1} = - \frac{Q}{{S\varepsilon_{0} \varepsilon_{r1} }}$$

2 $${\text{Inside Dielectric 1}}:E_{2} = - \frac{Q}{{S\varepsilon_{0} \varepsilon_{r2} }}$$

The two materials start to separate from each other, when the external applied force is released, producing a potential difference (v) in the external circuit. The potential difference between the electrodes is depicted as Eq. 3

3 $$v\left( t \right) = E1d1 + E2d2 + Eairx$$

The V-Q-x relationship is described by putting the ρ into Eq. 1 and is shown as Eq. 4

4 $$V\left( t \right) = - \frac{Q}{{S\varepsilon_{0} }}\left( {\frac{{d_{1} }}{{\varepsilon_{r1} }} + \frac{{d_{2} }}{{\varepsilon_{r2} }} + x \left( t \right)} \right) + \frac{\sigma sc}{{\varepsilon_{0} }} x \left( t \right)$$

The open circuit voltage (V_oc_) can be derived from Eq. 4, as the current becomes zero at open circuit condition as shown in Eq. 5:

5 $$Voc\left( t \right) = \frac{{\sigma {\mathrm{sc}}}}{{\varepsilon_{0} }} x \left( t \right)$$

In addition, the short circuit current and transferred charge can be demonstrated as:

6 $$Q_{sc} = \left[ {\frac{{S\sigma_{sc} x \left( t \right)}}{{\left( {\frac{{d_{1} }}{{\varepsilon_{r1} }} + \frac{{d_{2} }}{{\varepsilon_{r2} }} + x \left( t \right)} \right)}}} \right]$$

7 $$I_{{sc}} = \left[ {\frac{{{\mathrm{S}}\sigma _{{sc}} \left( {\frac{{d_{1} }}{{\varepsilon _{{r1}} }} + \frac{{d_{2} }}{{\varepsilon _{{r2}} }}} \right)v\left( t \right)}}{{\left( {\frac{{d_{1} }}{{\varepsilon _{{r1}} }} + \frac{{d_{2} }}{{\varepsilon _{{r2}} }} + x\left( t \right)} \right)^{2} }}} \right]$$

In most of cases, TENG devices is connected with external load resistance and the output performance can be estimated as:

8 $$V = IR = R\frac{dQ}{{dt}}$$

The operating mechanism of the TENG devices can be better expressed by Maxwell displacement and expanded Maxwell equations. The fundamental form of Maxwell’s equations is shown below:

9 $$\nabla \times D = \rho_{f} \left( {Guass \,Law} \right)$$

10 $$\nabla \times B = 0$$

11 $$\nabla \times E = - \frac{\partial B}{{\partial t}} \left( {Faradays\, Law} \right)$$

12 $$\nabla \times H = J_{f} + \left( {\frac{\partial D}{{\partial t}}} \right) \left( {Ampere \,Maxwell \,Law} \right)$$

The fundamental form of Maxwell equations is applicable to time-independent dielectric mediums. A significant challenge is shown in the case of time-dependent dielectric medium or in the case of moving media. This problem was figured out by Prof. Z. L. Wang in 2017 and 2022 in some of his articles [37, 38]. To solve this problem, Prof. Z. L. Wang proposed an expanded mathematical equation for moving charge media systems or time-dependent dielectric mediums [39, 40]. The expanded Maxwell equation's form is obtained by including polarization density (*Ps*) associated with the displacement vector. In this scenario, electrostatic charges arise on the medium surfaces owing to the triboelectrification effect, giving rise to the fundamental concept of TENGs. The modified displacement vector is represented as:

13 $$D = \varepsilon_{0} E + P + Ps = D + Ps$$

where ε_0_E is the external field, Ps is the added polarization term due to surface triboelectric charges. Hence, the modified form of the Maxwell is described as:

14 $$\nabla \times D = \rho_{f} - \nabla { } \times Ps \left( {Guass \,Law} \right)$$

15 $$\nabla \times B = 0$$

16 $$\nabla \times E = - \left( {\frac{\partial }{\partial t} + v \times \nabla } \right) B \left( {Faradays \,Law} \right)$$

17 $$\nabla \times H = J_{f} + \left( {\frac{\partial }{\partial t} + v \times \nabla } \right) \left( {Ps + D} \right)\left( {Ampere \,Maxwell \,Law} \right)$$

where *v* shows the movement velocity of the medium.

In conclusion, the fundamental form of Maxwell equations is related to fixed/static medium surfaces, while the expanded Maxwell equation is associated with time-dependent mediums. Figure 2c depicts the comparison study between the Maxwell equation for moving and stationary mediums. Hence, the Maxwell equation in its expanded form comes up with the basic concept of nanogenerators.

### Materials Selection and Their Classification for TENG Devices

**Fig. 3:**
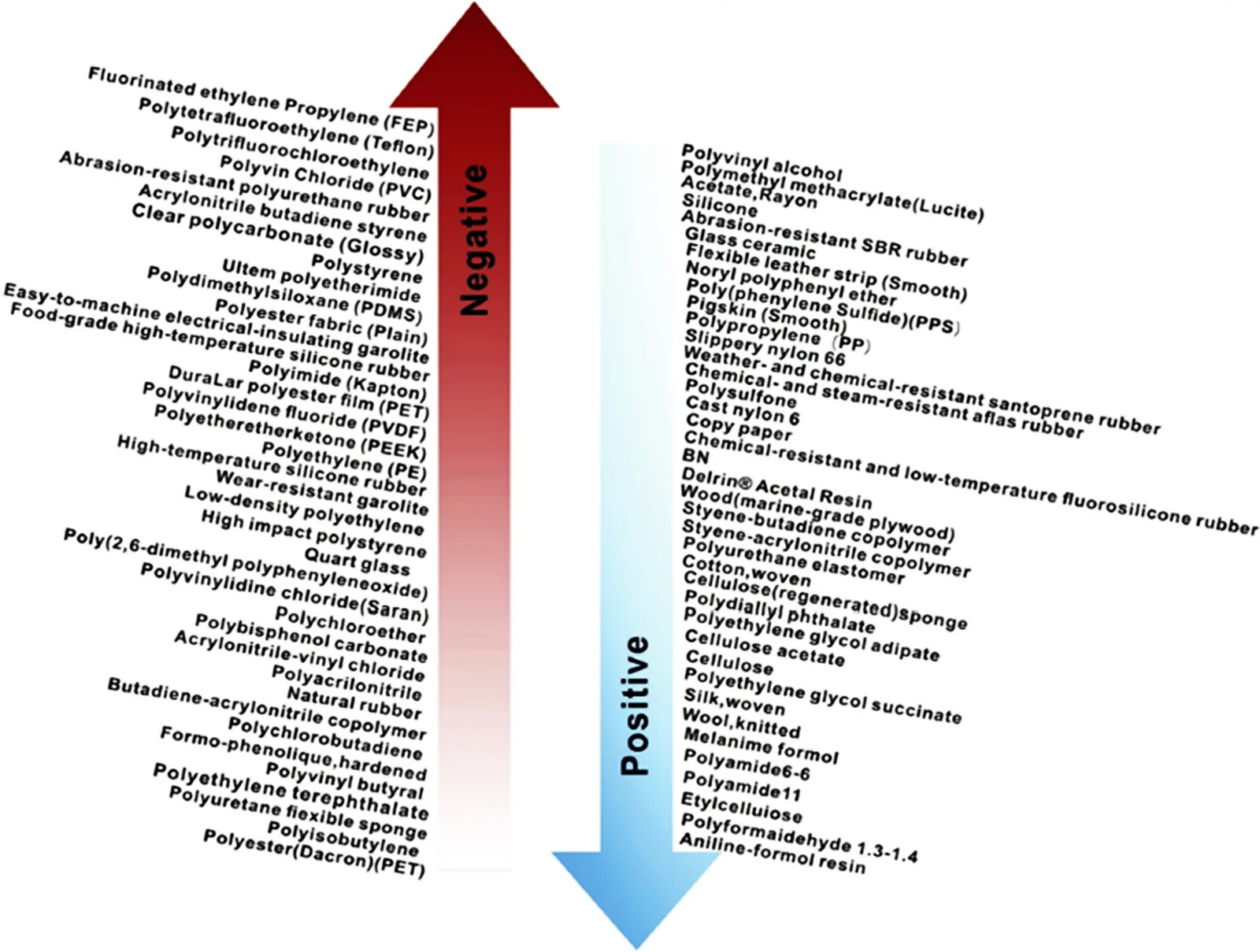
A fundamental series of the tribo-positive and tribonegative materials for the fabrication of TENG devices, reproduced with permission from Wiley, Copyright@2020 [41]

According to the working mechanism of TENGs, the material's polarity, which indicates its ability to lose or gain electrons, is directly related to the charge generated during the contact separation in the triboelectric phenomena [41]. Materials that lose or gaining electrons during triboelectrification produce a potential difference leading to an electrical energy output when they move toward or separate from each other. Various researchers have attracted great attention by exploiting the effect of the polarity of various materials on TENGs’ output performance. Several studies have been published that examine the polarity of various materials and arrange them from the most negative to positive tribo-polarity to construct the triboelectric series [41] displayed in Fig. 3. The polarity of the materials depends on chemical composition and functional groups attached to the surface. Materials that possess greater electronegative groups have a larger ability to gain electrons and generate a higher output power [42]. Generally, fluorinated-containing materials, such as polytetrafluoroethylene (PTFE), polyvinylidene fluoride (PVDF), and fluorinated ethylene propylene (FEP), are considered to be electronegative materials [43]. In addition, the materials containing amino groups, such as polyethylene terephthalate (PET), polyamide (PA), and many other nitro-functional groups-based polymers, are considered electro-positive [41]. These aforementioned materials have a remarkable ability to gain or lose electrons during triboelectrification. Additionally, materials such as 2D materials [44], metal–organic frameworks (MOFs) [45, 46], ferroelectric polymers, and flexible composites have been reported for preparing TENGs based on electronegativity and polarity to optimize/enhance triboelectric performance. Plenty of research has been published on various triboelectric materials, such as flexible/stretchable materials [20], 2D materials [47], self-healing polymers, bio-waste [48–50], and so on, to enhance/maximize the energy efficiency of TENG/self-powered devices.

The self-powered nanogenerators/sensors are essential for spacecraft and satellite applications. The triboelectric materials utilized in self-powered nanogenerators/sensors for spacecraft and satellite applications are chosen based on their polarity, durability, and performance under harsh environmental conditions. The most suitable materials for the space application include polymers (PTFE and PDMS) and composites, demonstrating a higher charge density through the triboelectric effect. The researchers have verified that PTFE has a very high corrosion and aging resistance under space environments conditions such as vacuumed and temperature variations. In addition, the PTFE has very strong elasticity and strength under cryogenic conditions [47–50]. The researchers are trying to improve the performance of self-powered triboelectric sensors for space applications by investigating novel polymers and composites that can lead to excellent durability and charge density against harsh environmental conditions in outer space. In summary, picking up the right materials for satellites and space applications is necessary for excellent energy efficiency and remarkable durability in the extreme environments of space.

### Recent Application Trends of TENGs

Since 2012, TENG has been highlighted as a promising energy harvesting technology based on triboelectric effect and electrostatic induction [51]. TENG was first reported by Prof. Z.L. Wang in 2012 and is considered a promising solution to harness mechanical energy owing to its high power density, simple structure, cost-effectiveness, and no materials restriction. The research area in the field of TENG has been extended toward its real-time applications, such as self-powered sensors [52], power supplies for robots [42–44], self-powered sensors for spacecraft, wearable devices for space suits, and human–machine interaction [45–47], owing to its exceptional advantages. Additionally, several articles have been published on TENG technology and have dramatically increased since 2012 [59]. Ongoing research of the TENGs aims toward systemization and industrialization [49–51]. Thus, various methods have been developed for the commercialization of TENG devices, such as power management, and these methods can show a promising solution toward commercialization and sustainability.

**Fig. 4:**
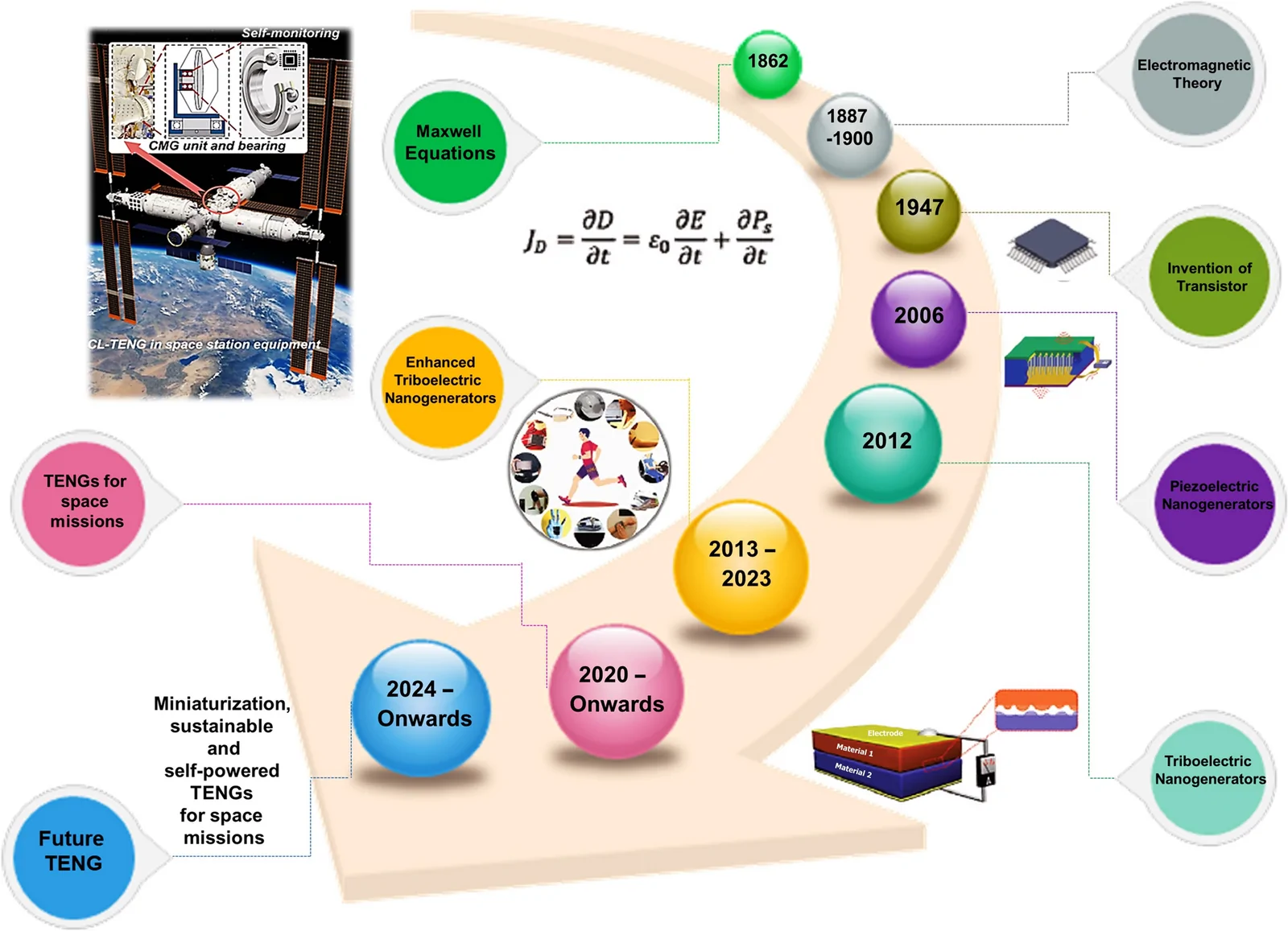
Roadmap toward the recent trends of the TENGs

Prof. Z.L. Wang, who discovered this TENG technology, has proposed a roadmap of the TENG devices over the previous ten years [63, 64]. According to proposed roadmaps, some researchers have attempted a tremendous effort to commercialize the TENGs. As a result, the research on the TENG has dramatically increased in the previous 10 years, and it can be expected that its advancement will be enhanced in the next 10 years for several commercial applications (Fig. 4). Recently, one of its application areas is space applications such as spacecraft, space satellites, and space suits [65]. Self-powered triboelectric sensors have been developed in recent years for the on-orbit health/structural monitoring of space systems [66], space object perception, and impact monitoring/safety of space stations. This review paper aims to analyze the development of TENG devices by discussing their utilization in space systems like satellites and space missions/crafts. This paper will summarize an overview of the self-powered triboelectric sensors for space exploration and satellite systems. Finally, we will discuss the TENG devices' future aspects and current challenges for satellites and space environments.

## Energy Harvesting for Space Missions and Satellite Systems

### Overview of Energy-Harvesting Technologies in Space Missions

**Fig. 5:**
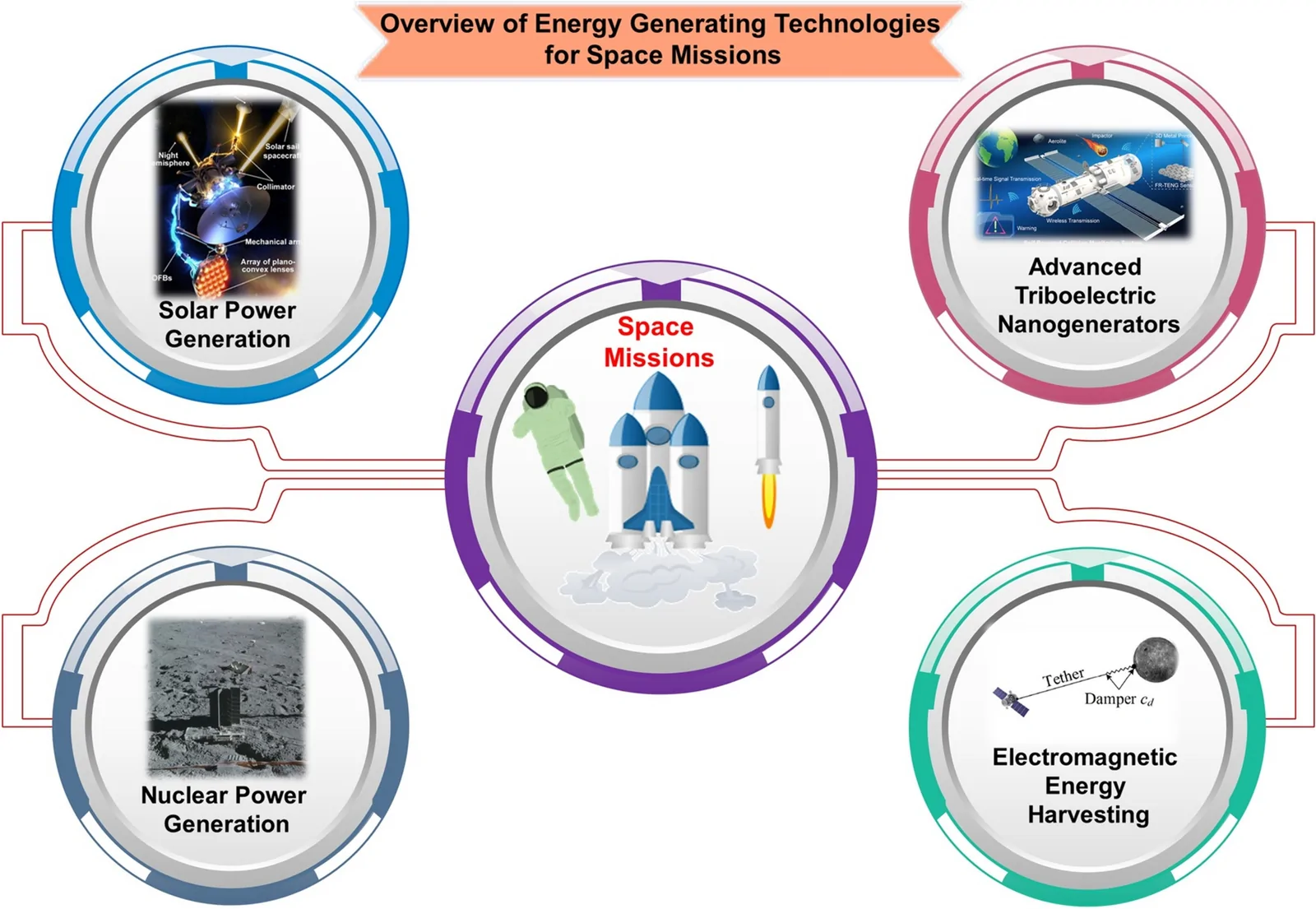
Overview of the energy harvesting technologies for the spacecraft/missions

Space missions and spacecraft usually depend upon energy-generating technologies for powering systems like scientific instruments and navigation. Several energy-harvesting technologies, such as photovoltaic solar panels, nuclear power generation, thermoelectric generators, and self-powered triboelectric nanogenerators, have been used to maximize the efficiency and sustainability of space missions/spacecraft. The energy-harvesting technologies mainly focus on the efficiency, durability, and sustainability of space systems and missions. Continuous research is ongoing in this area for the success of future space missions. Hence, this section will review the energy-harvesting technologies, such as solar power and nuclear power to function in the extreme conditions of outer space, as shown in Fig. 5.

#### Solar Energy Harvesting for Space Applications

Harnessing power is an important aspect of enabling spacecraft and related equipment to operate properly by utilizing the energy from the sun to generate electricity for different devices and tools onboard vehicles or systems in space. Solar panels are typically designed strategically in arrays to optimize exposure to sunlight while also being able to follow the sun’s path across the sky. Gallium arsenide (GaAs) and silicon (Si) are the most frequently used materials in these solar panels [67, 68]. These arrays can generate excessive power outputs of ~ 120 kilowatts (kW) for the International Space Station (ISS) and 110 watts for the National Aeronautics and Space Administration (NASAs) Mars Rover Perseverance. Modern solar cells are more effective than conventional panels, with higher energy efficiencies of 28% -30%, surpassing the conventional 15%-20% efficiency of solar panels. Special space-grade solar panels are being built to endure harsh space conditions lasting over 15 years despite potential performance declines (from cosmic radiation and micrometeoroid impacts). Solar power proves valuable for space missions; however, existing solar cells purely rely on batteries for energy storage. In addition to that point, spacecraft face power loss in areas and the potential risks associated with deploying solar arrays due to their complexity. Developing flexible solar panels with higher efficiency is a key priority in enhancing solar technology for space applications/missions.

**Fig. 6:**
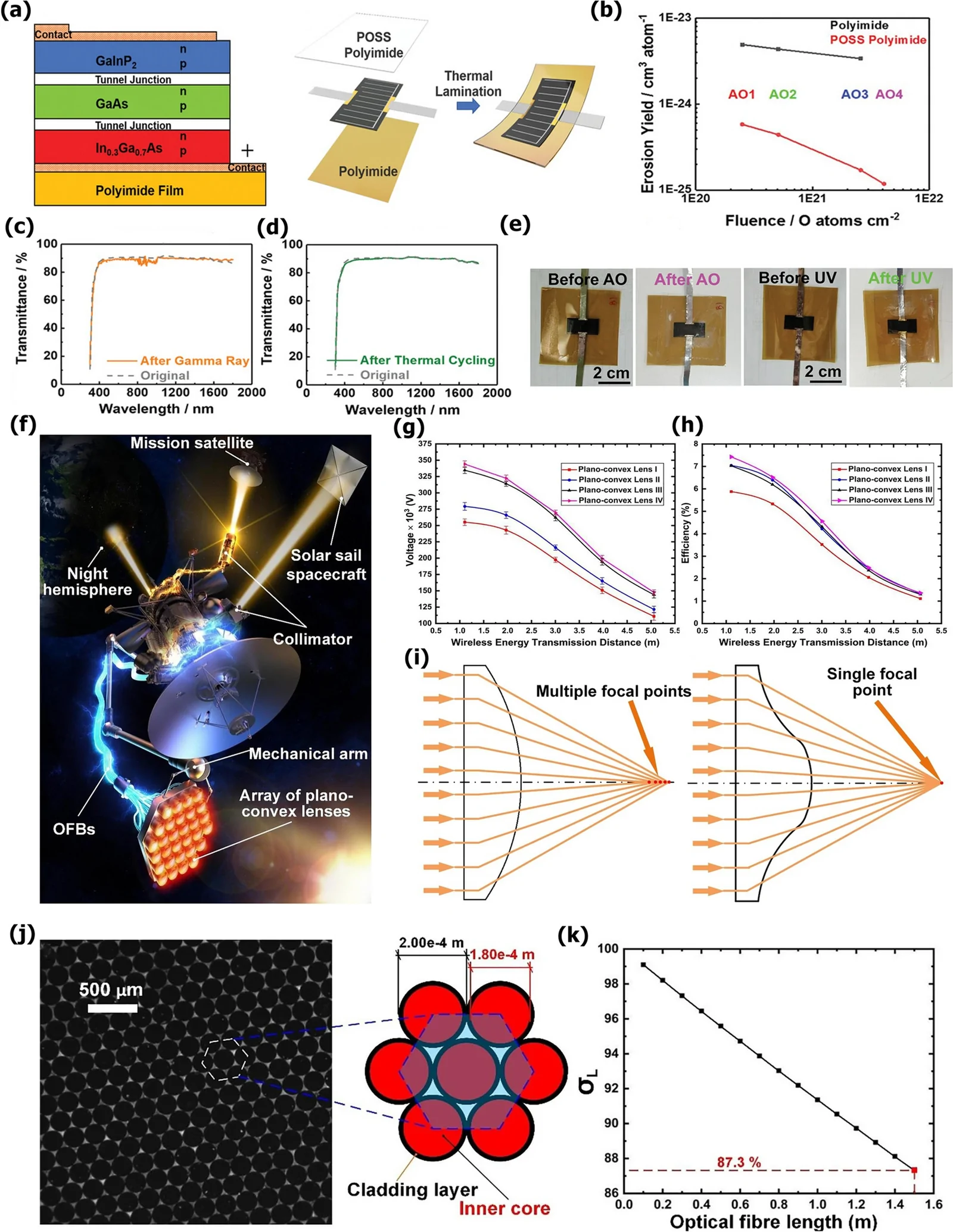
Solar energy harvesting for space applications.** a** Schematic of the POSS polyimide encapsulated and GaAs based solar cell (thin-film, triple junction); **b** erosion yields measurements of POSS polyimide films; **c** and **d** gamma ray exposures of POSS polyimide films; **e** pictorial images of the GaAs based solar cell before and after ultraviolet/ atomic oxygen exposure, **a-e** reproduced with permission from Wiley, Copyright@2021 [69]; **f** A conceptual schematic of the wireless energy transmission system based on concentrated sunlight through a single convex lens; **g** and **h** efficiency and voltage of the different convex lenses with the change in wireless energy transmission distance;** i** spherical aberration by aspheric lens and light condenser; **j** and **k** calculation of the energy transmission of the OFB and transmission efficiency with the variation of the OFB length, **f-k** reproduced with permission from Elsevier, Copyright@2022 [70]

Solar power stands out as an efficient energy source for space exploration; however, there are considerable hurdles that must be catered to in order to maximize its benefits effectively. In this regard, in 2021, Qian et al. developed a sealed flexible thin-film solar cell based on GaAs and polyhedral oligomeric silsesquioxane (POSS) polyimide for space applications [69]. The proposed flexible solar cells were fabricated with gallium indium phosphide (GaInP_2_) as a top cell, GaAs as a middle cell, and indium gallium arsenide (In_0.3_Ga_0.7_As) as bottom sub-cells with bandgaps of 1.89, 1.42, and 1.00 eV, respectively. All layers were deposited through the chemical vapor deposition (CVD) method, as shown in Fig. 6a. A top transparent POSS polyimide thin film and a bottom space Si adhesive/yellow polyimide thin film were laminated to create a sealed flexible triple-junction GaAs thin-film solar cell [69]. The new adaptable solar panels exhibited highly stable performance with a density of 4.1 × 10^21^ atoms cm⁻^2^ when exposed to atomic oxygen (AO) and endured 89.5 ESH (Equivalent Solar Hours) under ultraviolet light (UV) exposure while achieving an energy conversion efficiency of 28.44%. Figure 6b shows that the erosion yields of POSS polyimide films reveal a reliable erosion yield of 4.90 and 4.33 × 10⁻^24^ cm^3^ atom⁻^1^ upon AO_1_ and AO_2_, with complete erosion after AO_3_. The effect of the thermal cycling and various exposures such as gamma rays etc., was also evaluated, as shown in Fig. 6c, d. The solar panels showed efficient results when exposed to gamma rays and thermal cycles; meanwhile, the impact of space radiation on POSS films was showcased with regards to UV exposure and gamma rays as depicted in Fig. 6e. Use of lightweight polyimide POSS and GaAs-based solar cells for both high and low orbits in space seems to be encouraging news for the future developments in space missions [69].

Researchers are working toward planning space missions that focus on asteroids and spacecraft formations run by power. There have been discussions about energy transmission systems connecting Earth with satellites through lasers, microwaves, and sunlight. However, remaining challenges must be overcome, such as energy provision and miniaturization for spacecraft formations powered by energy and wireless energy transmission systems. To overcome these problems, in 2022, Teng et al. developed a wireless energy transmission system based on concentrated sunlight through a single convex lens to harness a single corresponding optical fiber bundle (OFB) and space sunlight for flexible power transmission [70]. The schematic of the CSEWTS is shown in Fig. 6f. The convex lens on the resource satellite captures and concentrates sunlight, which then travels through the optical fiber to a collimator, wirelessly powering the solar sail spacecraft. The coupling effect between the optical fiber bundle and the convex lens was investigated using four different Plano-convex lenses, with the calculated efficiency and voltage for wireless energy transmission shown in the results of Fig. 6g, h. The major differences between the four Plano-convex lenses is in terms of their geometrical optics (which define light convergence) and coating (which affects light transmission). The spherical aberration and aspheric aberration that can focus the light at a small point and different points are shown in Fig. 6i. Moreover, the energy transmission calculation and efficiency were calculated as shown in Fig. 6j, k. Solar energy emerges as a contender for space operations and expeditions thanks to its performance and capacity to deliver power in demanding conditions. Nevertheless, overcoming challenges (associated with solar energy) such as fluctuating radiation levels (around 1361 W m^−2^ in space), panel deterioration, and efficient heat regulation is important. Hence, it is necessary to innovate flexible and eco-self-sustaining power solutions to guarantee a seamless energy provision for electronic gadgets and sensors in space settings.

#### Nuclear Power Harvesting for Space Applications

Nuclear energy sources like RTGs play a significant role in powering modern-day space systems when solar energy is not enough to meet the energy demands of spacecraft and exploration vehicles during several space missions like Mars or other similar outer reaches of our planetary system where sunlight is scarce [71]. Nuclear energy offers additional advantages of providing high energy density (~ 24,000 to 30,000 W-hours per kilogram), reliability (99.99%), and longevity (~ 10 to 20 years). This enduring energy source is generated by converting heat produced through the decay of isotopes (such as Plutonium 238) into electricity. Electrical energy produced through nuclear energy sources is essential for supporting life functions onboard space missions for communication devices, scientific instruments, and internal heating systems. These nuclear energy sources offer several advantages, especially in harsh space conditions (extreme temperatures and intense radiation levels) where most conventional energy sources cannot operate properly. These RTGs play an important role in ensuring the success and sustainability of extended space expeditions due to their dependable performance and lasting durability. RTGs provide a power source that is not severely influenced by factors like dust or orbital movements that are common challenges of space environments. As space missions are expanding and efforts to explore our universe are increasing, nuclear energy sources will remain vital for advancing human progress and supporting complex scientific research.

**Fig. 7:**
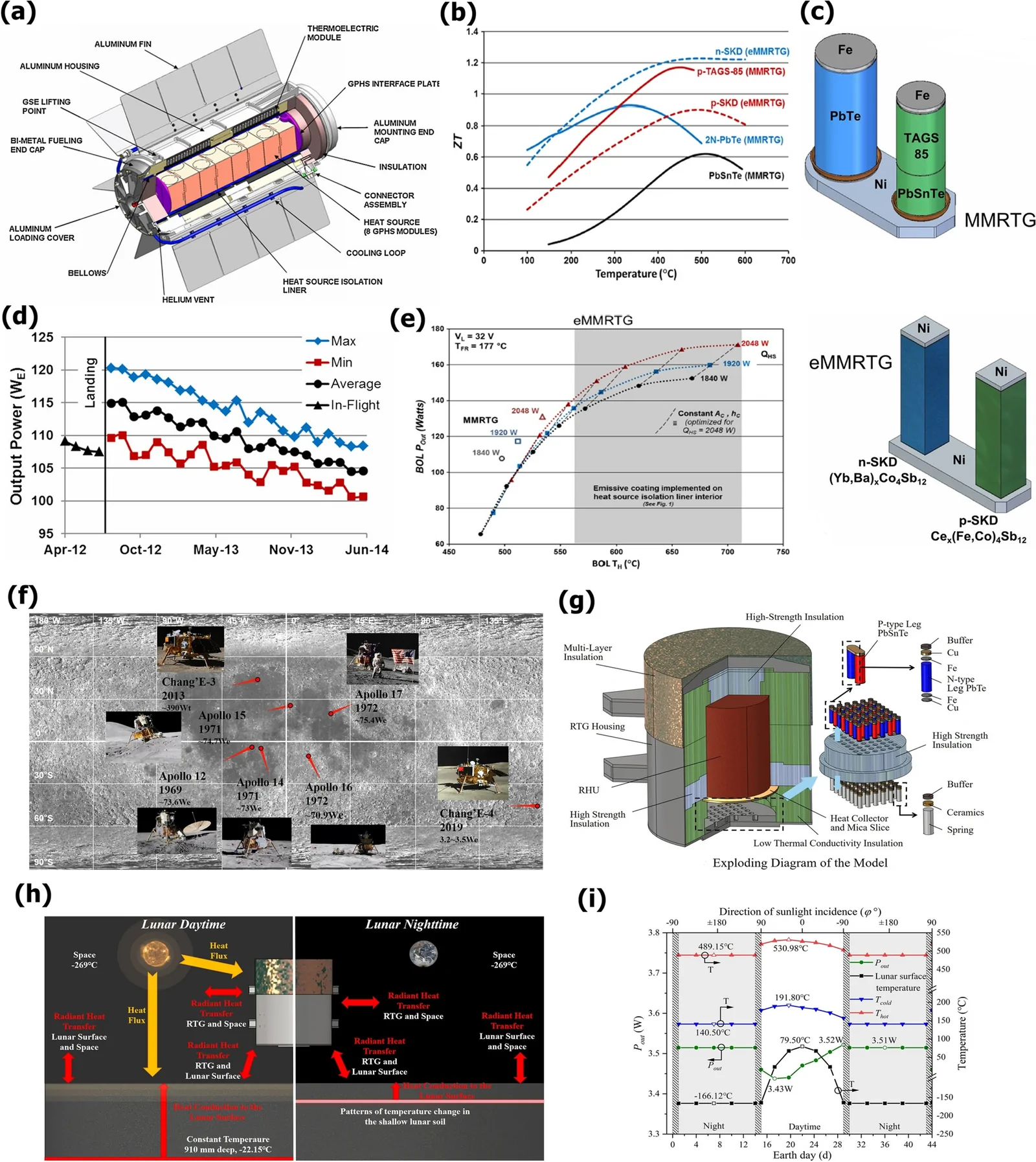
Nuclear energy harvesting for space applications. **a** Schematic overview of the side view of the MMRTG, **b** thermoelectric properties of the MMRTG and eMMRTG, **c** eMMRTG and MMRTG couples, **d** output power density of the MMRTG during the first year (Martian), **e** output power as function of the hot-side temperature, **a–e** reproduced with permission from Elsevier, Copyright@2015 [72]; **f** the utilization of the RHU/RTG on the lunar surface, **g** the model diagram of the Chang’E-4 RTG in the simulation environment, **h** the schematic overview of the boundary conditions (lunar surface), and **i** the output performance of the Chang’E-4 RTG in an overall lunar day, **f-i** reproduced with permission from Elsevier, Copyright@2023 [73]

Spacecraft missions have utilized nuclear power propulsion including Mars rovers (e.g., Curiosity 2011 and Mars 2020), lunar and Mars landers (Surveyor Series)), crewed outposts (Lunar Gateway, NASA's conceptual Mars Base Camp, ISS, and Project Orion), deep space orbiters (Voyager 1 and Voyager 2 launched in 1977, Cassini-Huygens-launched in 1997 that lasted till 2017, New Horizons-launched in 2006 and lasted till 2015, and Mars Reconnaissance Orbiter-MRO), ocean world landers (Titan Submarine-NASA, Europa Clipper-NASA, and Ocean Worlds Lander-NASA), and robotic probes (Cassini). The two primary types of nuclear power technology are RTGs and Fission Power Systems (FPS). FPS generates electricity through controlled fission reactions of heavy atoms like Uranium-235 (U-235) or Plutonium-239 (Pu-239), providing significant energy (kilowatts to megawatts) in space [23]. In 1965, the U.S. launched a 43-day FPS space mission, but it was premature and ended early [23]. Subsequent attempts in the 1980s and 2000s by the Department of Energy (DOE) and NASA to develop FPS missions failed due to high costs and complexity[23]. In contrast, RTGs generate electricity from the natural decay heat of Plutonium-238 (Pu-238), producing up to 1 kilowatt energy. Since 1969, NASA has launched various RTGs space missions, including Voyager, Apollo, Pluto New Horizons, and Mars Curiosity. The latest RTGs mission, Cassini (launched in 1997), used the Multi-Mission RTG (MM-RTG), which produces 110 watts, enhanced by various n- and p-type skutterudite materials for improved efficiency. Hence, a demonstration of MMRTG (eMMRTG) based on Skutterudites (SKD) materials is shown in Fig. 7a, while the thermoelectric properties of this proposed RTGs are shown in Fig. 7b along with the thermoelectric couples of the MMRTG in Fig. 7c [72].

The output power of both MMRTG and eMMRTG is shown in Fig. 7d, e, respectively. Hence, SKDs are prime candidates for RTGs. Tailin et al. developed and investigated the performance of the Chang'E-4 RTG based on a thermoelectric model in laboratory and lunar surface environments for the first time [73]. Various space missions based on the RTG/Radioisotope Heater Unit (RHU) on the moon are shown in Fig. 7f. Chang'E-4 RTG has two operating modes, including a short-circuit mode and a power supply mode. Moreover, the Chang'E-4 RTG was also simulated in a COMSOL environment, and a schematic of the model in the simulating environment is shown in Fig. 7g. The simulated results showed that various factors can affect the RTG performance in the lunar surface environment. At the same time, these results also helped to explore the effect of RTG placement orientation and the angle of influence of the sun on thermoelectric performance. The boundary conditions of the lunar nighttime and daytime during the simulating environment are shown in Fig. 7h. The calculated results of the RTG model deviate from reported values by less than 4%, while the performance of the total lunar day is shown in Fig. 7i. Overall, maximum power output can be obtained when the Chang'E-4 RTG are placed horizontally during the daytime (facing sunlight) [73]. This allows them to function on their own for a prolonged time. This makes nuclear power technology an option for spacecraft missions because it is dependable, has a lifespan, and has a high energy capacity.

Apart from the mentioned positives of using nuclear energy as a power source for space crafts, there are certain associated negatives with nuclear energy sources, including thermal management and safety concerns. The first and foremost drawback of using nuclear energy sources like RTGs is their radiation emissions posing severe hazards to spacecraft equipment and crew members' health during missions. Moreover, the advanced technology and high-profile materials involved in the construction and manufacturing of RTGs make them expensive, with costs often surpassing $1 million/unit (because of the utilization of isotopes). RTGs provide ~ 100 watts of power, which is significantly lower than the ~ 120 kilowatts generated by solar panels on larger spacecraft. Dealing with nuclear materials requires safety measures of the highest standards for transportation and their proper disposal is a non-negotiable factor that adds much responsibility, mental stress, and complexity to overall regulations of space missions. Sometimes, an unwanted and unforeseen mishap can occur during the launch of a space mission, which could result in nuclear substance release into the environment, leading to direct safety concerns. Even though RTGs can last for extended periods (~ 10 to 20 years), the decline in their power (due to decay) imposes constraints on how long they can sustain missions effectively and once these nuclear energy sources enter in space, it becomes difficult to either modify or enhance their ability, hence, limiting their flexibility to adapt to evolving mission requirements or technological advancements. These obstacles need evaluation when devising missions on nuclear power sources.

Integrating TENGs into space missions can potentially help to handle these drawbacks of high cost (exceeding $1 million per unit), limited power output (100 watts), and safety concerns (arising from their radiations and inflexibility post-deployment) that are associated with nuclear energy. TENGs provide a lightweight renewable power alternative capable of harvesting energy from surrounding mechanical sources, like space vibrations, with efficiency levels reaching ~ 28% to 30%. TENGs are designed to be small, flexible, sustainable, and robust, making their integration into spacecraft setups easy without adding much weight to the overall system. The modular structure of TENGs allows for combining units to meet energy needs without using hazardous radioactive materials. Additionally, TENG systems can be adjusted according to advancements and specific space mission requirements, enabling upgrades during ongoing missions, thus improving its overall effectiveness and viability for space expeditions.

#### Other Energy Harvesting Techniques for Space Applications

Energy harvesting is the basic concept of converting ambient energy into a useful form through renewable energy resources [74]. State-of-the-art research in energy harvesting devices showed that these devices are a viable source of energy for various electronic and automotive space industrial devices such as sensors, pressure sensors, and fuselage sensor networks in aircraft. The conventional energy harvesting techniques/devices used for space devices are photovoltaic energy harvesters and nuclear power energy harvesters [75]. Apart from conventional energy sources for space missions, several other unconventional methods could also be used as energy sources to improve sustainability and efficiency. TEGs were capable of converting temperature variances by using the contrast between sunlight and heat and the coldness of space to produce electricity, which was ideal for long-term missions that require a constant power source [76, 77]. Radioisotope power systems (RPS) could utilize the heat generated from decaying isotopes like plutonium 238 to offer energy supply for many years. This technology has been successfully employed in missions such as the Mars Curiosity rover and Voyager probes, establishing their durability over time. Energy can also be harvested by utilizing energy from vibrations to power sensors in dynamic environments effectively [71, 78]. Another source of energy in space missions could be electromagnetic energy harvesting, which involves the conversion of radio waves and solar radiation into electricity through antennas to support communication devices across space systems [79]. Biomechanical energy harvesting is also another type of unconventional energy harvesting system for space missions that aims to convert the movements of astronauts or robots into power to potentially improve technologies. Chemical energy harvesting methods like fuel cells can make use of resources on planets such as Mars to generate electricity and reduce the need for fuel transportation.

**Fig. 8:**
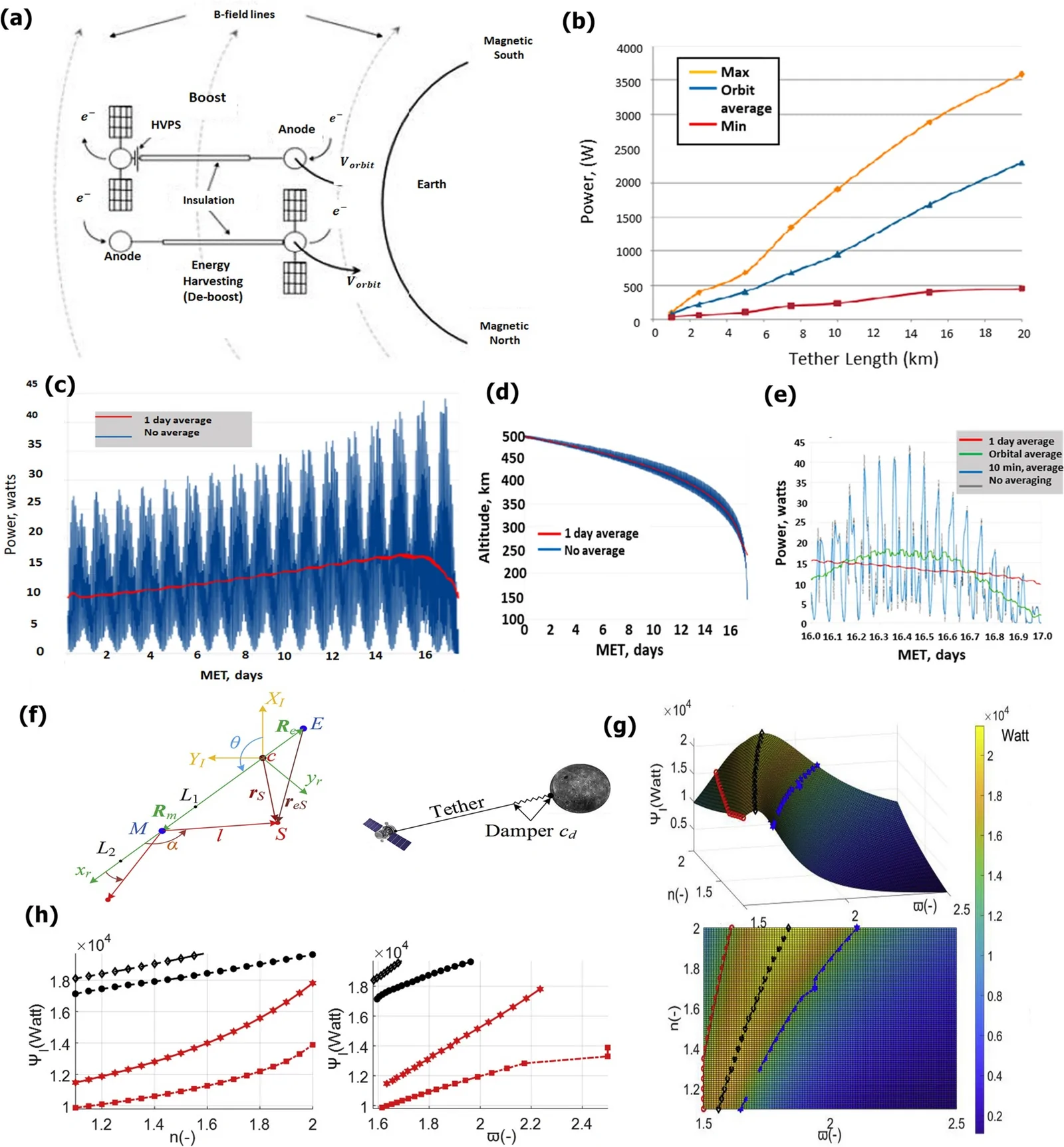
Other energy harvesting techniques for space applications, **a** Electrodynamic tethers that can be utilized as an energy harvesting device**, b** power output variation generated by an Electrodynamic tether, **c** output power as a function of elapsed mission time, **d** altitude as a function of elapsed mission time, **e** power and various averages as a function of elapsed mission time, **a-e** reproduced with permission from AIAA, Copyright@2010 [80]; **f** Space tether and elastic tether at a base of system, **g** Output power based on analytical methods, and **h** the peak points of the output power from positive Ψ and ᶯ directions respectively, **f–i** reproduced with permission from Elsevier, Copyright@2019 [82]

Electrodynamic tethers (EDTs) and plasma charging systems are innovative energy harvesting methods to harvest the wasted energy in orbital environments [80]. EDTs can be utilized to produce the power onboard spacecraft orbiting the planetary orbits (earth) and harvest the spacecraft’s orbital energy. The main advantage of the EDTs is to generate a significant power level (3.8 kW) when other conventional sources are not available [81]. In this regard, Bilén et al. demonstrated electrodynamic tethers to harvest the energy for space platforms and propulsion on various scales such as large-to-small, nano-, and pico-scales [80]. The EDTs-based energy harvesting mode/boost mode is shown in Fig. 8a. The generated power, orbit average, and peak variation increase as tether length increases, as shown in Fig. 8b. The power was calculated across load resistor as a function of mission elapsed time (0.558 kg, 1,300 m, aluminum tether) and is shown in Fig. 8d, e. Moreover, the altitude of the EDT harvesting device (with satellite) a function of mission elapsed time was measured as shown in Fig. 8c. As in orbit around the planetary objects, the power is not consistent, so it is necessary to measure the time frame for which the power will be required. Hence, the amount of harvested energy varies based on tether length, type, and plasma contactor design. EDT systems having small sizes can harvest 50% more energy as compared to solar panels. Further, in 2018, Liu et al. developed a space-tethered system based on resonance phenomena for lunar orbital energy harvesting [82]. These combined approaches could present a useful resource for unconventional energy harvesting during space missions. The tether with negligible mass is attached to the moon surface along with a damper at its base is shown in Fig. 8f. The power output based on the analytical method is shown in Fig. 8g. The peak point curves of the power output are shown in Fig. 8h. Hence, the space tethered systems attached to the Moon's surface can easily harvest the energy from the mechanical damping of the tether as it experiences elongation motion due to time-varying tidal forces.

### Overview of TENGs in Space Environments/Missions

TENGs’ lightweight, high output power and simplest design make them easier to integrate into space systems (spacecraft and satellites) without adding significant weight. TENGs can also work efficiently in harsh environments, which is necessary for long-term space missions. The researchers attempted to utilize it for space missions and satellite systems after being inspired by its advantages/self-powered abilities. In this section, we have summarized current research, developments, and applications of the TENGs in space environments and missions.

#### TENGs for Planetary Exploration Missions

Planetary exploration missions such as Mars and moon required a durable and self-powered energy solutions owing to the harsh environmental conditions such as wide temperature ranges and high radiations. This section highlights role of TENGs under simulated Martian environments to harvest mechanical energy from wind, vibrations, and dust interactions.

Exploring Mars through space missions has significant importance for the survival of the human race. Perseverance rover and Mars Science Laboratory (Curiosity) are examples of missions that are being used to study the geology of Mars’ climate and also to look for any evidence of previous life. The Perseverance rover is specifically engineered to hunt for ancient microbial life and gather samples that could be brought back to Earth in the near future [83, 84]. Mars has a day length of approximately 24.6 h, which is similar to Earth and could be beneficial for human settlement on Mars [85]. The existence of water ice at the poles and underground reservoirs is crucial for facilitating upcoming expeditions and settlements on Mars. Moreover, the 95% presence of carbon dioxide (CO_2_) in the Martian atmosphere poses both hurdles and prospects for creating methods to generate oxygen (O_2_), which is a must for human life. Studying the climate and geology of Mars yields knowledge about how the planet has evolved and the likelihood of extraterrestrial life existing elsewhere in the universe. Mars is located about 225 million kilometers from Earth, and it is a key destination for developing technology for long-term space travel and exploration that could lead to unraveling mysteries of our solar system.

Energy requirements have the highest significance in exploring the likelihood of extraterrestrial life elsewhere in the universe. Solar cells on Mars face several limitations, including reduced sunlight intensity, as Mars receives only about 43% of the solar energy (as compared to Earth), significantly impacting panel efficiency [86]. Frequent dust storms can cover solar panels, blocking sunlight and necessitating complex cleaning mechanisms. In 2008, the Phoenix Mars Lander failed, and the performance of rovers degraded due to dust covering their solar panels. Additionally, seasonal variability can lead to fluctuations in energy production that can affect the operations of space missions. Mars also has a slightly longer day (approximately 24.6 h) and long nights, requiring energy storage solutions that complicate the design of power systems. Moreover, temperatures can drop to − 125 °C, hence reducing solar cell efficiency. In contrast, nuclear energy systems, such as RTGs, have high initial costs that can exceed $1 million per unit and pose radiation risks also to nearby equipment and personnel. The use of nuclear materials involves stringent regulatory requirements, complicating mission planning. RTGs provide continuous power (around 100 watts), but their output diminishes over their 10–20-year lifespan due to radioactive decay that potentially limits their long-term mission viability. Furthermore, launch failures pose risks of releasing radioactive materials, raising safety concerns. Both energy sources present unique challenges that must be addressed in Mars mission planning.

The limitations of conventional energy sources (solar and nuclear) certainly require an alternate energy source (TENGs) that could overcome these limitations. TENGs show potential as energy harvesting tools engineered to function in the distinctive environment of Mars by transforming mechanical energy from natural sources like wind and vibrations into electrical power. TENG devices excel in such conditions while capturing mechanical movements to generate energy for powering scientific equipment and supporting exploration vehicles and living spaces. In this regard, in 2017, M-L Seol et al. developed a Mars analog weather chamber to analyze the effect of environmental factors on the output of the TENG device in the Martian environment [11].

**Fig. 9:**
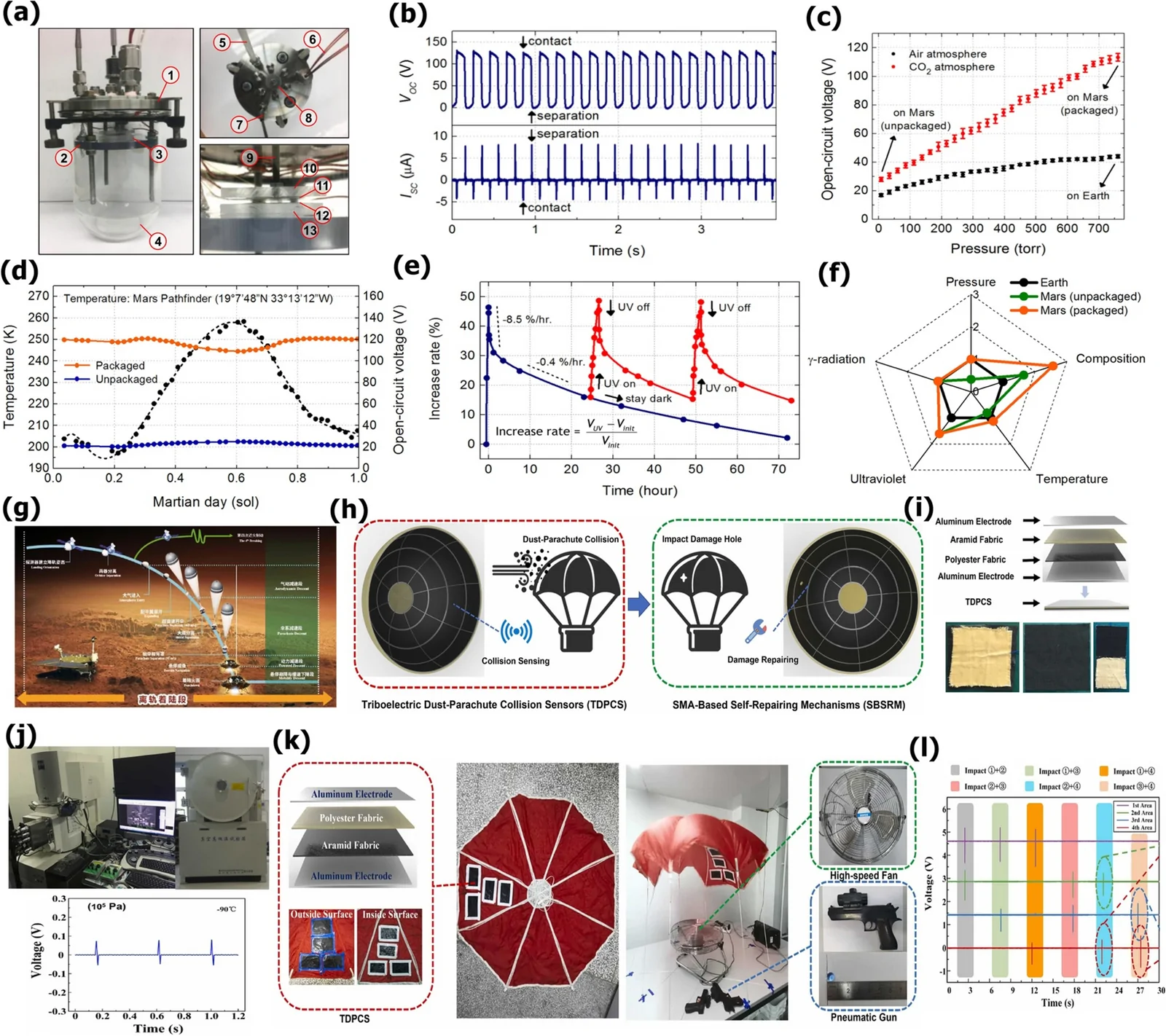
TENGs for planetary exploration missions (Martian environments).** a** Pictorial image of Mars weather chamber’s configuration, **b** voltage and current of the measured in Mars weather chamber, **c** output voltage under different atmospheric pressures such as air and CO_2,_ **d** variation in the temperature as a function of Martian day and output voltage under unpacked and packaged conditions, **e** discharging curves in sunny and dark weather conditions, **f** comparison of the output performance on the Mars with Earth’s, **a-f** reproduced with permission from Elsevier, Copyright@2017 [11]; **g** the landing of Zhurong Rover, **h** the parachute for the Martian dust environment, **i** schematic of triboelectric dust-parachute collision sensors (TDPCS), **j** vacuum operating platform to see the effect of the Mars environment on the TDPCS and output result at − 90 °C, **k** evaluation of the output performance of the impact monitoring systems on TDPCS, and **l** output performance of the double shot test, **g–l** reproduced with permission from Elsevier, Copyright@2022 [87]

The Mars analog chamber contains a metal lid and bell-shaped jar, as shown in Fig. 9a. The TENG device was mounted on the acrylic stage that was suspended in the middle of the chamber, as shown in Fig. 9a. The metal lid features transmission lines for providing linear vibration input energy and monitoring output energy and chamber conditions, while also controlling ambient conditions [11]. The TENG receives mechanical input energy from an electromagnetic shaker. The electrical measurements of the TENG, which has an area of 9 cm^2^, demonstrated excellent reproducibility under a constant input force of 28.7 kPa, as shown in Fig. 9b. The effect of ambient conditions such as atmospheric pressure (0.6 kPa-0.06% of Earth's atmospheric pressure at sea level), and temperature (-125 °C during winter at the poles to approximately 20 °C near the equator during the day in summer with an average temperature of around − 63 °C across the planet) of the Martian environment on the TENG output performance was examined. The open circuit voltage of the TENG device under various atmospheric pressures, such as air (8 torr) (40 V) and CO_2_ atmosphere (760 torr) (110 V) was measured, and the obtained results are shown in Fig. 9c. The voltage was higher in both air and CO_2_ atmospheres, with CO_2_ yielding a greater voltage 157 times than air. Additionally, the wider temperature ranges on Mars were measured to evaluate their effect on TENG performance, as shown in Fig. 9d. Low daily performance change implies that TENG has the proper characteristics to pair with solar panels, which have severe performance degradation at night. Moreover, the effect of the UV light on the TENG performance was also examined while keeping the other parameters constant according to the Martian environment, as shown in Fig. 9e. A 254 nm wavelength was used that is present in abundant form the Martian surface. The UV light is set to be present during day hours while its presence is less during the night hours. Therefore, discharging behavior during the night time was also measured, as shown in Fig. 9e. These charging and discharging behavior suggest that exposure to UV radiation greatly boosts the efficiency of TENG devices when used on Mars’s surface. These TENG devices perform best during the day when exposed to UV rays and show a decrease in output (30% open circuit voltage) at night but still above the initial levels. Hence, the TENG can work efficiently in the Martian environments, as shown in Fig. 9f.

Researchers are working on enhancing parachutes to streamline the Entry Descent Landing (EDL) phase of missions to Mars as space technology progresses swiftly. In the Martian environment, the regolith particles could travel up to several kilometers, as shown in Fig. 9g. Various tests have been tested for the parachute for Mars missions, but the effect of the dust particles was not investigated. In this regard, in 2022, Ding et al. developed a reliable parachute with Shape Memory Alloy (SMA) and triboelectric dust-parachute collision sensors (TDPCS) based self-perceiving system for the harsh dust storm Martian environment [87] as shown in Fig. 9h. The monitoring system includes the TDPCS to identify collisions between parachute fibers and dust particles efficiently. This innovative smart system utilizes shape memory alloys (SMAs) to alleviate pressure in regions with damaged holes [87]. These TDPCS are embedded within the parachute material to analyze damaged areas and monitor the dust impact, as shown in Fig. 9i. The TENG sensor was fabricated using polyester and aramid as triboelectric materials, while Aluminum foils served as electrodes with dimensions of 40 mm × 40 mm × 2 mm. TDPCS was encapsulated with insulating tape to avoid the effect of environmental factors such as humidity, etc. The performance of the TENG was evaluated as related to the Mars environment, as depicted in Fig. 9j. From these measured results (voltage), it can be concluded that TENG sensors can be operated efficiently in the Mars environment. Additionally, impact monitoring tests were performed through TDPCS on real parachutes. The 4 TDPCS were attached to the parachute prototype and are demonstrated in Fig. 9k. Different type of signals was generated through a double shot test, as demonstrated in Fig. 9l. Although the parachute fabric is under strong vibration due to airflow and shot impact, the performance of TDPCS is reliable and sensitive, which proves the impact area perceiving function of this monitoring system [87]. Additionally, yang et al., proposed a novel self-powered TENG designed for dust removal from solar panels under Martian conditions [88]. This study investigates the dust removal based on TENG devices for various wind speeds and panel tilt angles, and experimentally verified its effectiveness in a Martian environment. In summary, TENG-based sensors or energy harvesting units showed a viable route toward planetary exploration missions such as Martian space exploration missions.

#### TENGs for Spacecraft's Design and Structural Health Monitoring

This section shows the integration of TENG devices in spacecraft design and structural health monitoring. In this section, we have discussed the research works including astronaut monitoring and bearing-integrated TENGs for spacecraft flywheel diagnostics. These novel works discuss the reliability and compatibility of TENGs in improving spacecraft performance and safety.

As space missions become more intricate and demanding of systems for health monitoring purposes, the reliability of systems is paramount. Power and weight budget issues are critical in spacecraft design. Scientists are developing highly efficient, cost-effective devices to provide continuous power for structural health monitoring. Conventional techniques face certain limitations to extreme conditions in space, including optical fibers (overweight, fragility, temperature sensitivity, installation complexity, high cost, and signal loss), piezoelectric sensors (limited frequency response, environmental sensitivity, calibration requirements, and nonlinear response), and accelerometers (sensitivity to noise, drift over time, limited range, and power consumption). In this context, TENGs are well-suited as self-powered devices for on-orbit health and structural monitoring of space systems. TENG devices are gadgets that transform energy generated by vibrations and movements into electrical power [89]. This feature of TENG devices renders them well-suited for monitoring the structural integrity and health conditions in space missions. TENG devices can harvest energy from sources surrounding them, such as vibrations stemming from rocket liftoff and the strain experienced by structures, becoming an uninterrupted power source for sensors used in monitoring without the need for external batteries. They produce voltages ranging from 1 to 10 V and deliver power outputs of up to 10 watts per meter cube for a variety of uses [90]. Their light and compact structure enables incorporation into spacecraft without adding any considerable amount of weight, which is an essential consideration for space missions. Additionally, TENG devices operate efficiently in challenging environments such as temperature of − 125 °C and atmospheric pressure of 0.6 kPa, making them well-suited for space missions. Using TENG devices to monitor stress levels and detect strain and damage in real time enhances structural stability and improves mission safety during space exploration.

**Fig. 10:**
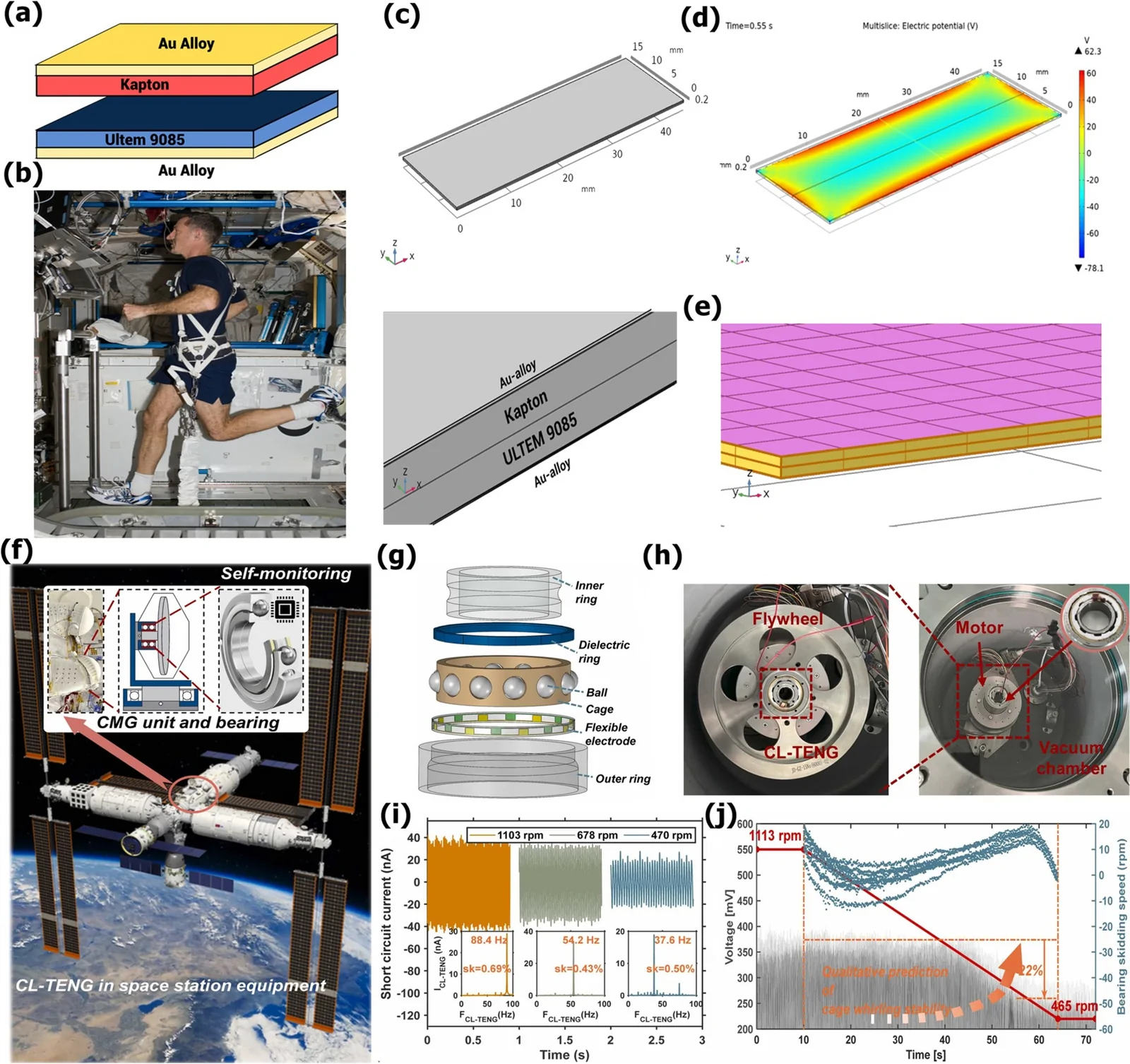
TENGs for spacecraft's structural and health monitoring: **a** Schematic of proposed a Phase-A design TENG, **b** pictorial image of the Astronaut runs on treadmill, **c** top view and layers of the TENG device in COMSOL environment, **d** the simulated result, **e** Mesh utilized for TENG simulation, **a–e** reproduced with permission from Elsevier, Copyright@2020 [91]; **f** schematic diagram of the self-powered CL-TENG utilized in the CMG unit of the spacecraft, **g** 3D schematic view of the CL-TENG, **h** testing chamber/environment for flywheel components integrated with CL-TENG, **i** output and FFT calculations of the CL-TENG under space conditions, and **j** output under acceleration and deceleration process, **f–j** reproduced with permission from Elsevier, Copyright@2024 [66]

In 2020, Ventre et al. proposed a Phase-A design TENG for various space applications, such as structural health monitoring through software simulations [91]. The TENG device was fabricated using Au alloy as an electrode, while Kapton and Ultem-9085 were utilized as triboelectric layers as shown in Fig. 10a. These proposed TENG devices should be utilized on the treadmill during Astronauts’ exercise time, as shown in Fig. 10b. The TENG sensor was designed using COMSOL software and dimensions of the modeled TENG is shown in Fig. 10c. The simulated results of the TENG sensor is shown in Fig. 10d, e. The maximum value of the voltage was 98 V at the vertical displacement of 250 μm, which indicates that TENG sensors could be suitable for monitoring astronauts' health. Later on, in 2024, Gao et al. proposed a lightweight TENG device for the spacecraft flywheel system's health monitoring [66]. The disturbance in a spacecraft's flywheel system arises from two factors: (1) vibrations at the bearing's fundamental train frequency and (2) skidding between the raceways and rolling elements [66]. Regular measurements are necessary to monitor micro-vibrations in the flywheel systems. The schematic diagram of the lightweight and compact (CL-TENG) for the flywheel system of the spacecraft with the sensing ability of the cage whirling is shown in Fig. 10f. The three-dimensional (3D) structural view of the bearing-based CL-TENG is demonstrated in Fig. 10g. It comprises a PTFE dielectric ring, a cage rolling bearing, and flexible interdigital electrode systems. The CL-TENG was tested in a space environment to monitor the flywheel's behavior under nonstationary conditions, with an outer ring-guided CL-TENG installed on a field motor-flywheel system. These experiments were performed in the pressure-negative chamber, as demonstrated in Fig. 10h. The short circuit current of the bearings was measured at three different speeds (1105, 678, and 470 rpm) and is shown in Fig. 10i. The time–frequency analysis of the open circuit voltage of the CL-TENG was measured under acceleration and deceleration conditions as shown in Fig. 10j. During the deceleration test, average speed decreased from 1113 to 465 rpm (voltage: 6–3 V) within 52 s, while during the acceleration test, average speed increased from 793 to 1113 rpm (voltage: 4.4–6 V) within 22 s. Hence, cutting-edge developments in self-powered triboelectric sensors offer promising solutions for on-orbit monitoring of spacecraft flywheel systems, providing a robust method for assessing flywheel health.

##### TENGs for Manned Space Equipment

Manned space mission requires wearable gadgets that can work under harsh conditions, while maintaining astronaut safety, mobility, and situational awareness. This section introduces the integration of TENGs into manned space equipment such as space suits and gloves for the continuous self-powered monitoring of physiological activity and astronauts body motion.

TENGs are advanced energy-harvesting technology with significant potential to enhance space suit capabilities in increasingly complex and longer-duration space missions. These exploration missions demand self-power solutions within space suits to function efficiently while maintaining sustainability. TENG devices have the proven ability to transform typical physical movements of astronauts (like walking or changing body positions) into electricity to keep wearable gadgets powered continuously [92]. These TENG devices can produce voltages from 1 to 10 V and generate power outputs up to 10 watts per meter cube [93, 94]. These electrical output values are enough to enable the operation of essential sensors and communication tools for life support installed in space suits without relying on batteries that ensure lightweight spacesuit design. These innovative TENG devices are not only lightweight and compact but also versatile in operation over a wide and extreme temperature range (from − 125 to + 70 °C) [95, 96]. Additionally, TENG devices can withstand extreme conditions, including radiation exposure and vacuum environments, ensuring reliable performance essential for astronaut well-being and safety. Real-time tracking of body signals and surrounding elements, through TENG innovation, has the potential to transform space gear significantly by boosting its self-sufficiency and supporting human space journeys in a more sustainable manner.

**Fig. 11:**
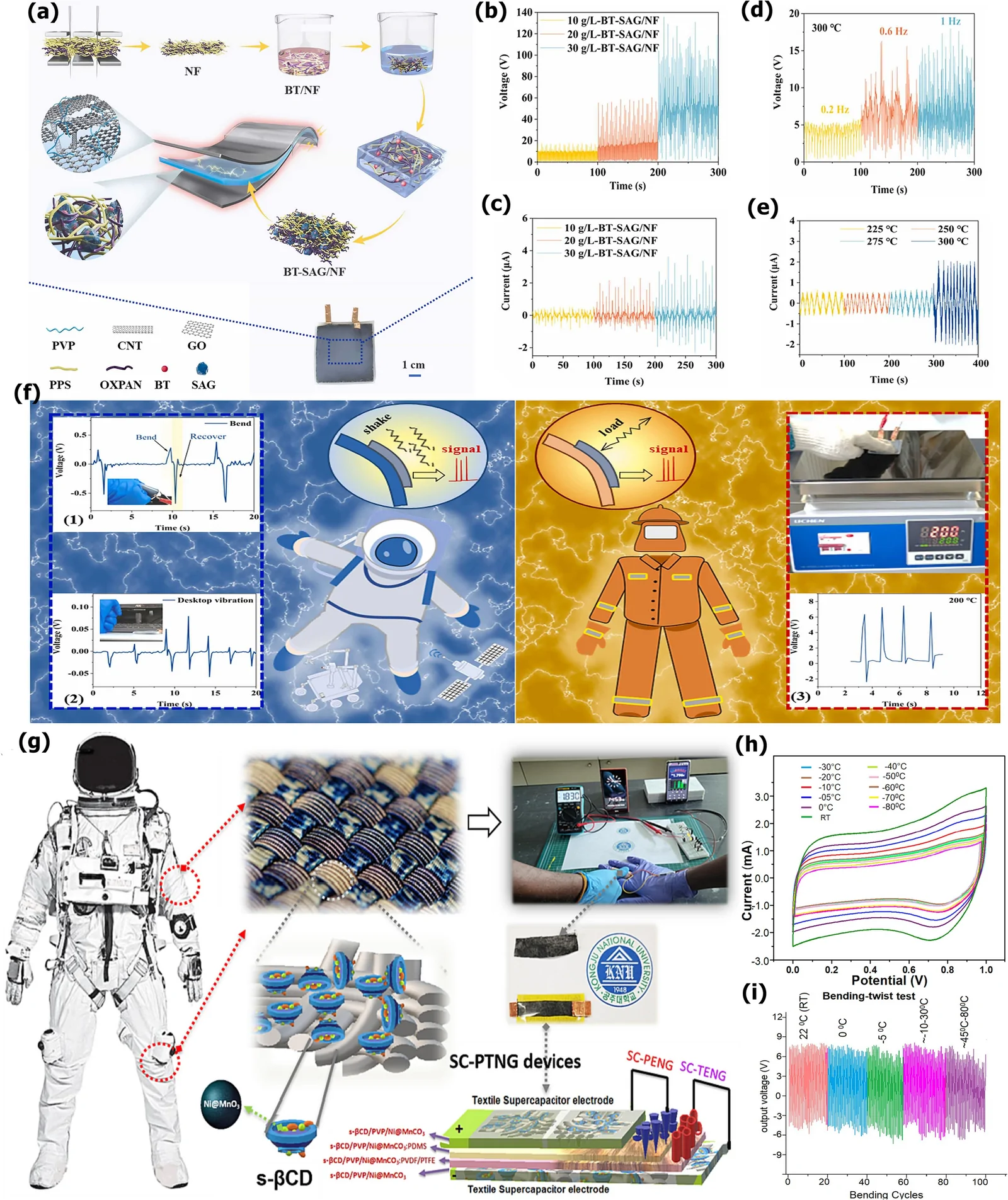
TENGs for Manned space equipment (space suits): **a** Schematic diagram of the preparation process of the TENG for various temperatures,** b** and **c** output performance of the BT-SAG/NF-based TENG devices, **d** and **e** output performance of the BT-SAG/NF-based TENG devices at high temperatures,** f** pictorial image of the driving the diode bulbs, **a–f** reproduced with permission from Elsevier, Copyright@2024 [97]; **g** textile-based PTNGs with super-capacitor device for space suits, **h** cyclic voltammetry curves of super-capacitor device at different temperatures, and **i** output voltages at 3.5 mm bending radius at different temperatures, **g-i** reproduced with permission from Elsevier, Copyright@2024 [65]

In this regard, in 2024, Jiali Hu et al*.* demonstrated a flexible aerogel-based, highly efficient TENG under diverse temperature conditions for space suits [97]. Figure 11a shows the preparation process of TENG devices for diverse temperature environments. The TENG was fabricated using a high-temperature resistant substrate (Silicon aerogels (SAGs)/needled felt (NF)). Moreover, the BaTiO_3_ (BT) particles were blended in SAGs to enhance the output performance of the TENG devices. First, the electrical performance of the high-temperature resistant silicon aerogel and fiber felts-based TENGs (HTFs-TENG) was evaluated at 25 ℃ by blending the various concentrations of BT particles as shown in Fig. 11b. The output performance (voltage and current) of HTFs-TENG increases with the enhancement of BT particles as shown in Fig. 11b, c. The voltage and current of the HTFs-TENG increased due to the enhanced relative permittivity of SAGs after adding BT particles. Subsequently, the output performance was evaluated at high temperatures (300 °C) to assess its self-powered capability for space suit applications. At high-temperature conditions, the voltage and current of the HTFs-TENG decrease and then slightly increase at 300 ℃ as shown in Fig. 11d, e. Thus, HTFs-TENG can respond to various temperature conditions, making it a suitable candidate for space suits and probes. It can generate signals at both low (25 °C) and high (200 °C) temperatures [97]. Moreover, this proposed device even produced signals by tapping a desktop near it and also in flexible conditions, as shown in Fig. 11f. These results prove that the self-powering nature of the proposed TENG device can be a suitable candidate for the space suits and probes due to its wide application temperature range [97]. Moreover, Zheng et al., developed a thermally stabilized TENG operating from − 29 to 400 °C for space suits [98]. In this work, polyacrylonitrile (TS-PAN) was utilized as tribonegative materials while mica film as tribopositive materials. Thus, proposed TENG device opens a new avenue for space wearable applications. Additionally, *Selvam *et al*.* proposed a cyclodextrin fabric textile-based tribo-piezoelectric nanogenerators (PTNGs) along with flexible supercapacitors for wireless wearable sensing devices under extreme environments [65] as demonstrated in Fig. 11g. The proposed hybrid self-chargeable power generator showed stability of 50,000 cycles and an excellent electrochemical performance at low-temperature ranges (0 to − 80 °C) as shown in Fig. 11h. The output voltage of the PTNG was evaluated at low temperatures as shown in Fig. 11i. Hence, this proposed hybrid system can be suitable candidate to meet the demands for wearable devices that can be equipped in low temperatures in space suits and also for wireless wearable devices [65].

**Fig. 12:**
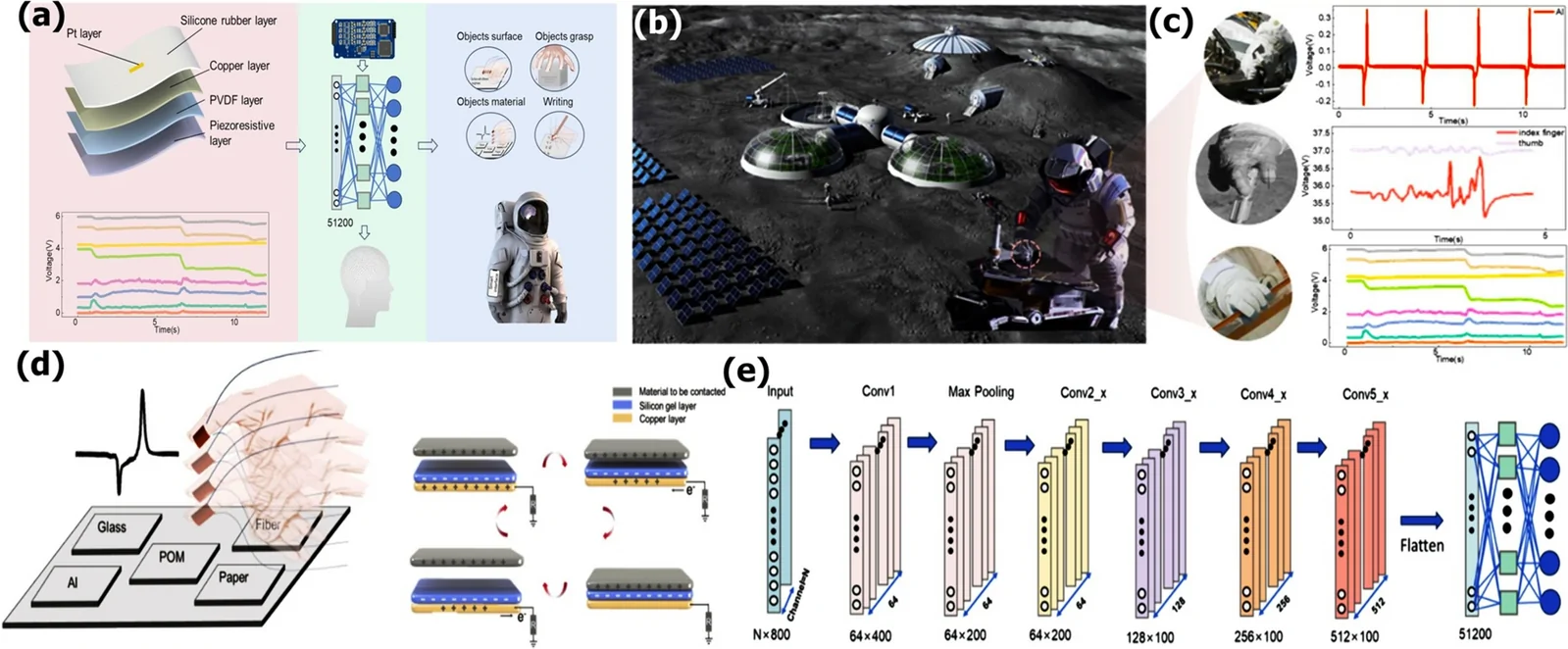
TENG-based tactile sensors smart extravehicular activities of astronauts (space gloves).** a** schematic of the design of the biomimetic tactile sensors (multilayered) sensors designed for a complex and highly sensitive touch perception, and **b** schematic of tactile signal diagram generated by biomimetic tactile sensors, **c** multi-finger tactile sensing extravehicular activities, **d** schematic diagram of one-finger tactile perception for material identification, and **e** structure of deep learning models for generated triboelectric signals, **a–e** reproduced with permission from Elsevier, Copyright@2024 [92]

Although some advancements have been made in the case of the piezoelectric sensor, temperature sensor, and tactile sensors. Still, it is challenging to demonstrate a multimodal tactile perception based on the triboelectric effect for astronaut activities that can enhance the multi-finger operations of astronauts. In this regard, in 2024, Kong et al. developed a tactile sensor (multimodal) mounted on space gloves for the multi-finger operations of astronauts [92]. A biomimetic tactile sensors (multilayered) sensor was designed for a complex and highly sensitive touch perception, inspired by the human fingertip's ability, as demonstrated in Fig. 12a. The proposed tactile sensor is composed of five layers that mimic how the skin senses touch based on piezoelectric, triboelectric, piezoresistive, and thermosensitive mechanisms. Further, a biomimetic perception intelligence system was proposed by integrating the deep learning techniques with a tactile sensor, as shown in Fig. 12a. The sensor sends the captured signals to the hardware part and passes them to the deep learning module, similar to how fingertip sensations are sent to the brain. Figure 12b, c shows the haptic feedback signals from BMLTS and BIPS during complex tasks using one, two, or multiple fingers. These signals can help astronauts perform spacewalk tasks, highlighting the great potential of BMLTS and BIPS to provide haptic feedback for smart, multi-finger operations in space. Moreover, BMLTS was utilized to identify the object materials depends upon the triboelectric unit as shown in Fig. 12d. After that, a multi-channel ResNet18-1d model/algorithm was used to process and recognize these signals effectively for real-time material identification (Fig. 12e). Hence, this tactile perception system for complex hand movements is helping to develop better touch perception, which is necessary for astronauts to perform precise and smart tasks during spacewalks. Moreover, TENG devices can generate electrical power as well as real-time monitoring signal simultaneously based on synergistic mechanism [99–102]. These devices served as both a self-sustaining power unit and a real-time motion sensor without the need for external batteries. For example, triboelectric signals generate from bending walking that can both power electronics devices and be utilized for gait pattern recognition, and fatigue monitoring. This synergetic mechanism is particularly necessary for manned space equipment’s, where real-time physiological feedback and long-duration power autonomy are necessary. In summary, smart self-powered TENG sensors present a promising solution for future space missions owing to their efficient sensing abilities, surface exploration, environmental monitoring, and compatibility with harsh environments.

##### TENGs/Self-Powered Sensors for Aeronautical Systems

This section discusses the integration of TENG-based self-powered sensors and energy harvesters into recent aeronautical systems. It provides advancements in UAV flight actuator monitoring utilizing TENG displacement sensors, enhancing flight safety and autonomy. Additionally, it demonstrates wind energy harvesting using hybrid TENG-EMG systems, demonstrating the potential of TENGs in aerospace applications.

**Fig. 13:**
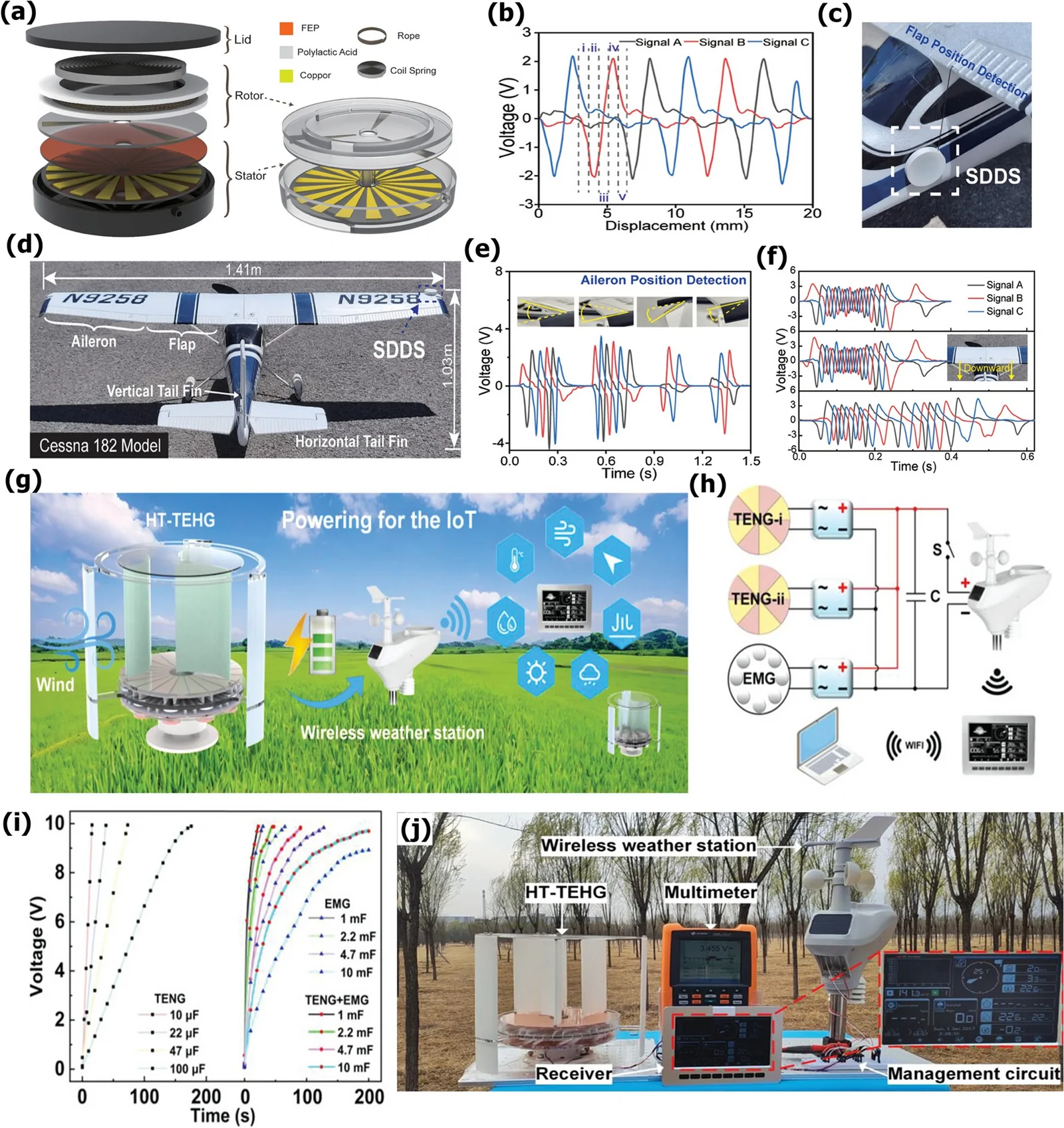
TENG devices/self-powered sensors for Aeronautical systems:** a** Schematic and electrode placement of SDDS based on a freestanding layer of TENG, **b** generated output signal of SDDS, **c** SDDS are attached on the fuselage to monitor the position of the flaps, **d** pictorial image of the Cessna 182 model, **e** output signals generated form ailerons of aircraft, **f** first two (synchronize) signals third (abnormal) signals during flap monitoring, **a–f** reproduced with permission from Wiley, Copyright@2023 [105]; **g** conceptual design of HT-TEHG, **h** circuit demonstration of application of self-powered weather station**, i** HT-TEHG was used to charge the various capacitors, and **j** experimental demonstration of TENG to supply the power for weather stations, **g–j** reproduced with permission from Wiley, Copyright@2024 [111]

The increasing demand for unmanned aerial vehicles (UAVs) in the industrial world is a serious concern about flight safety, particularly due to their limitations in flight attitude perception compared to manned aircraft. Various sensors are attached to the manned aircraft that can measure the airspeed efficiently, while UAVs depend on simple sensors like accelerometers that control the positions of surfaces like flaps and wings [103, 104]. In the case of manned aircraft, the existing position sensors can monitor the movement of important parts of the aircraft efficiently, while these sensors do not exist in the case of UAVs. Existing position monitoring solutions used in manned aircraft are incompatible with UAV systems, highlighting the need for new, specialized position sensing technologies to address these challenges [105]. To solve these problems, TENGs are suitable candidates for aerospace applications because they can produce electrical power from mechanical movements without external batteries. TENGs can work in extreme environmental conditions, such as various temperatures and higher altitudes, making them suitable for aerospace applications [106, 107]. Moreover, TENGs have self-powered capabilities that can harvest low-frequency energy, such as small movements in aircraft wings, and require less maintenance, making them suitable for aerospace and aeronautical systems. In this regard, in 2023, Zhou et al. developed a self-powered triboelectric displacement sensor to monitor the position status of the flight actuators in UAVs [105]. The self-powered digital displacement sensor (SDDS) based on a freestanding layer of TENG and grating structure is shown in Fig. 13a. This proposed sensor has been attached to the wing of a UAV, which is responsible for contraction and stretching along with the movement of the UAV flaps. The output signals were generated from the three sets of freestanding electrodes, as shown in Fig. 13b. The SDDS was further tested on a Cessna 182 model to validate its real-time applicability, as shown in Fig. 13c, d. Figure 13e shows the detected signals for aileron motion below and above the main airfoil. Finally, both flaps of the Cessna 182 model were synchronized, and two detected synchronized signals along the abnormal signal (Signal C) are shown in Fig. 13f. Hence, this system (SDDS) provides a potential route toward practical flight applications and ensures a certain level of flight safety [105].

Energy harvesting in aeronautical and aerospace fields can be accomplished by converting the ambient energy resources such as wind [18], vibration, pressure changes, and structural deformation during the flight. Various methods, including electromagnetic [108], thermoelectric [109], piezoelectric [110], and TENGs, convert these energy resources into electrical power. Among them, TENGs are suitable candidates for aerospace applications because they can produce electrical power from the environmental forces without external batteries. Zhu et al. hybrid triboelectric and electromagnetic nanogenerators (HT-TENG) based on bionic blade-shift for wind energy harvesting in a wide wind speed range [111]. The schematic diagram of the HT-TENG that can convert natural wind energy to electrical power for weather wireless stations, as shown in Fig. 13g. The hybrid nanogenerators combine a drag-type turbine and a bionic blade lift-type turbine to enhance aerodynamic performance and drive EMG and TENG independently. In addition, the working principles of the HT-TENG for powering the wireless weather station are depicted in Fig. 13h. Three power generators are connected in parallel to charge the capacitors, which then power the weather station, enabling self-powered remote weather monitoring. Figure 13i shows the charging analysis of HT-TENG at 5 m s^−1^ wind speed. The real-time application of the HT-TEHG was also demonstrated, as shown in Fig. 13j. The HT-TENG successfully supplies power for wireless weather stations and realizes self-powered meteorological information monitoring. Hence, TENGs can harvest wind energy efficiently, supplying electrical power to electronic devices, thus avoiding the inconvenience and high cost of transmission lines or disposable batteries.

##### TENGs for In-Orbit Robotic Operations/Collision Monitoring

This section demonstrates the demonstration of TENG devices for in-orbit robotic systems and safety monitoring for space missions. It demonstrates how TENGs provides a self-powered sensing in robotic platforms utilized for spacecraft assembly/terrain exploration, increasing autonomy and operational lifespan in harsh environmental conditions.

*TENG based Robotic Systems for In-Orbit Assembly:* Space exploration holds importance in today's research landscape and space-based robots contribute significantly to leveraging space resources through continuous technological progressions. The main tasks of these robots involve spacecraft upkeep and examination along, with identifying terrains in space [112–114]. Numerous nations are now directing investments towards in-orbit assembly technologies as seen in initiatives like NASAs Dragonfly project that focuses on enabling antenna assembly in space [115]. Nonetheless, despite these advancements, challenges such as risks, limited operational capacity harsh environments of space, and efficiency issues persist along with potential sensor performance challenges, in space conditions [116, 117]. Henceforth the demand arises for aerospace sensors that are lightweight, dependable, and economical. TENGs are transforming the way energy is harvested by offering power solutions for unique and diverse applications like space-traveling robots. These robots play an important role in exploring planetary surfaces by carrying out independent movements and data collection in harsh outer space conditions. TENG devices have the ability to harvest energy from the actions of robots (crawling or climbing) into electrical energy that enables the continuous supply of electricity for onboard sensors and propulsion systems. The small, autonomous, and portable structure of TENGs makes it easy to integrate them into space-traveling robots that can withstand the harsh environmental conditions of space. Space robots equipped with TENG devices can enhance their operational lifespan and autonomy by harvesting ambient energy from their surroundings. This allows them to perform a range of tasks, including but not limited to gathering soil samples and studying habitats, which paves the way for exploration and supports future human settlement efforts.

**Fig. 14:**
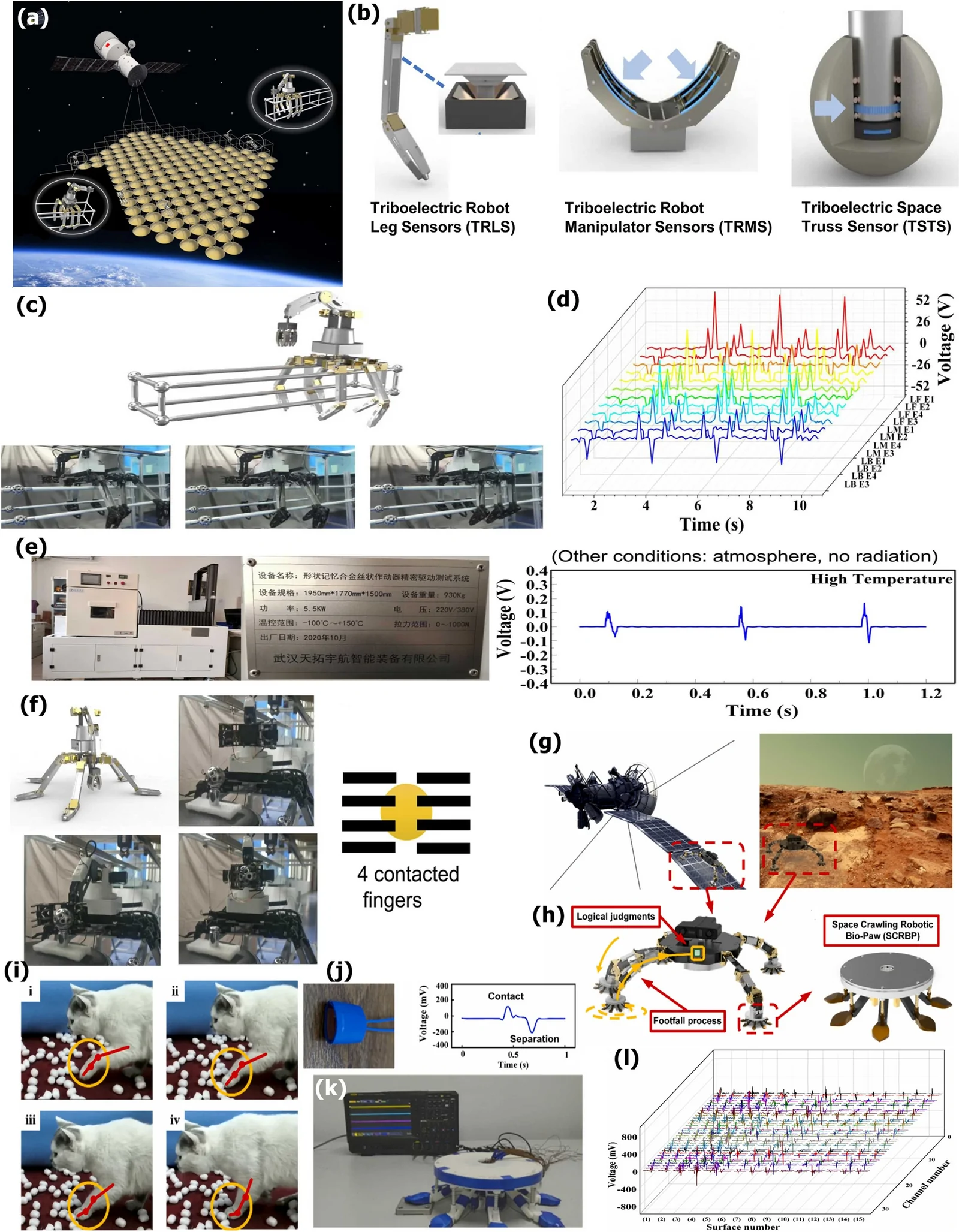
TENGs-based Robotic Systems for In-Orbit Assembly:** a** Schematic of robotic system attached or moved onto the surface of the spacecraft,** b** Assembly of the TENG sensors, **c** schematic climbing motion of six sensors of the robotic system, **d** output signals generated from the sensors, **e** experimental validation for the TENG in space environments and output performance at high temperatures, **f** pictorial images of the grabbing tests of TENG sensors, **a–f** reproduced with permission from Elsevier, Copyright@2022 [118]; **g** Schematic of space crawling robot in space environment, **h** schematic of the SCRBP, **i** step by step process of cat’s football process, **j** schematic and output of the TRCS, **k** pictorial image of the SCRBP, **l** output performance of the SCRBP to various objects, **g–l** reproduced with permission from Elsevier, Copyright@2023 [50]

In this regard, in 2022, Hou et al. developed a self-assembling/attaching robotic cluster system based on the triboelectric effect to monitor the state (crawling) of the robot during the in-orbit assembly process [118]. The schematic of the proposed robotic system that can be attached or moved onto the surface of the spacecraft is shown in Fig. 14a. This proposed robotic with two triboelectric sensors can monitor the safety and improve the efficiency of in-orbit assembly of large-scale spacecraft. The triboelectric sensor comprises of three major parts includes triboelectric robot leg sensors (TRLS), triboelectric robot manipulator sensors (TRMS), and triboelectric space truss sensors (TSTS), as shown in Fig. 14b. The TRLS sensors were investigated within a complete cycle, analyzing signals from one of the six legs since all legs exhibit the same motion pattern over time. The signals from the creeping gait, monitored across all sensors on the six legs of the S2A2RC system, are shown in Fig. 14c, d. Additionally, the effect of the harsh conditions of the space on the TENG sensors was also evaluated, as shown in Fig. 14e. The triboelectric sensors showed excellent performance at high (− 150 °C) and low temperatures (− 100 °C) within diverse and harsh environments of the space. Finally, the truss component grabbing tests were also investigated, as shown in Fig. 14f. Thus, this proposed system can overcome the current issues associated with previously reported sensors, such as cost, scalability, and energy consumption [118]. Owing to continuous development in aerospace sensor technology, Hou et al. demonstrated a cat paw-inspired space crawling robotic (SCRBP) for the identification of terrain surfaces [50]. Figure 14g depicts a space robot doing missions on big spacecraft or planets, with SCRBP placed on its end. Meanwhile, this proposed SCRBP consists of a touch sensing system along with self-powered triboelectric sensors that can deliver real-time information in multi-directional sensation [50]. Moreover, the SCRBP's structure is depicted in Fig. 14h. The SCRBP features eight groups of sub-paws, each consisting of a palm pad and toe pad that can independently contact surfaces. Additionally, it incorporates four different types of TENG devices located in various positions. The triboelectric sensors are divided into types based on their working mechanisms, such as triboelectric contact sensor (TRCS) and triboelectric sliding sensor (TRSS). One TRSS and three TRCS are attached to each sub-paw of the SCRBP. Figure 14i shows the football behavior of the cats that are placed on a carpet under a terrain surface. The schematic of the triboelectric contact sensor is shown in Fig. 14j, while the pictorial image of the SCRBP is shown in Fig. 14k. Furthermore, using machine learning methods, the SCRBP can effectively identify surfaces based on 15 features (Fig. 14l). The SCRBP shows promising capabilities for adapting to space environments and collecting surface data. These triboelectric sensors are light in weight, consume less power, and can function in challenging space conditions. Equipping space roaming robots with triboelectric sensors presents a viable option for space missions, improving effectiveness and dependability in surface exploration and orbital assembly techniques.

**Fig. 15:**
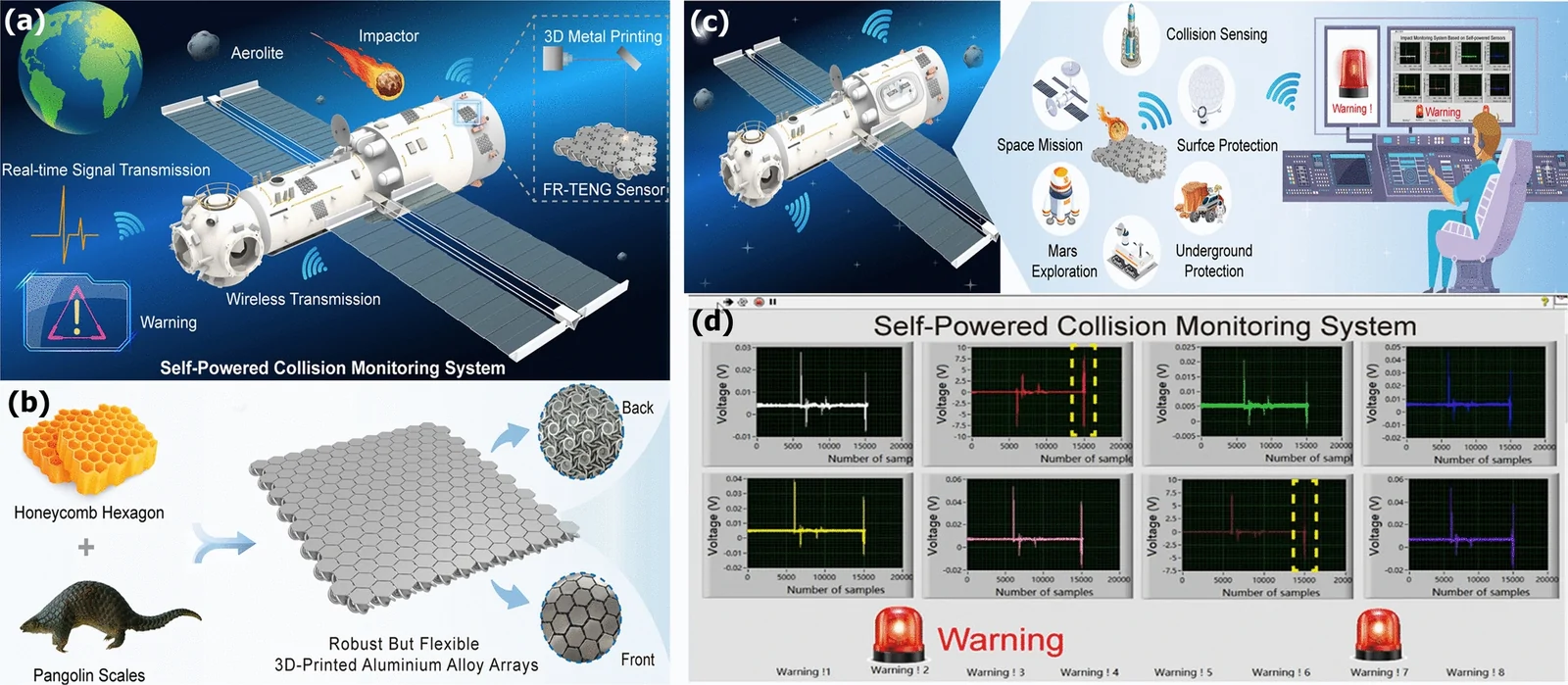
Self-powered TENG sensors for Space Debris Detection and Safety Monitoring** a** Schematic of the conceptual design of flexible FR-TENG array, **b** schematic overview of the structures of the pangolin and hexagonal scales, **c** conceptual overview of the FR-TENG-based wireless monitoring system, and **d** pictorial image of the LabVIEW system in the impact state, **a-d** reproduced with permission from American Chemical Society, Copyright@2023 [119]

*Self-powered TENG sensors for Space Debris Detection and Safety Monitoring: *The first artificial satellite was launched in 1957, marking the start of space missions. The presence of space debris has appeared a significant threat for the safety of future of space missions. However, the space debris problems became a serious concern as space missions progressed [119]. It comes from old satellites, discarded rocket parts, and fragments from collisions and explosions. The debris can create problems for satellites, active spacecraft, space stations, and bases in space [119]. In 2018, on the International Space Station (ISS), the astronauts observed a small decline in air pressure due to a tiny meteorite impact, which highlighted the dangers of space debris [120]. Therefore, space debris monitoring is necessary for the safety of space missions. Traditional technologies, including ground-based radar systems, cannot detect small debris and predict their paths, especially in areas with lots of debris. Moreover, energy consumption is also a major concern for the sensors during long space missions. Hence, there is an urgent demand to develop sensors and monitoring systems with less energy consumption as well as the capability of detecting small pieces of debris and improving space safety. In this regard, TENG devices offers viable route to demonstrate the self-powered, lightweight, and scalable sensing and actuation systems for autonomous space debris detection and cleanup [121]. In this regard, in 2023, Sun et al. proposed and designed a flexible, robust, and one-piece 3D printed TENG for the impact monitoring and safety of the space stations and outer space [119]. A self-powered collision monitoring system (SPCMS) was demonstrated, as shown in Fig. 15a. The SPCMS is composed of 3D printing technology along with self-powered TENG sensing. This proposed system can send alerts to astronauts on time, detect collisions, and monitor the space station's status after an impact [119]. The proposed devices have a quick response time, high stability, simple fabrication process, and small size as compared to previous traditional sensors. It has a hexagonal structure showing excellent stability, strength, and a simple fabrication process. Moreover, the surface of pangolin scales, with a hard and layered overlapping structure provides both self-protection and a degree of flexibility. Based on these aforementioned the 3D printed TENG device was proposed as demonstrated in Fig. 15b. This type of TENG remains stable under external pressure and impact like the hexagon, rigid but flexible like the pangolin scales, which can be attached to the curved area for monitoring in multi directions and protection. The TENG device has the advantages of flexibility and robustness that can directly integrate into the surface of the space station without any additional complexities. Therefore, a self-powered triboelectric sensor was developed that can show a wireless signal collision monitoring system to demonstrate its practical applicability as depicted in Fig. 15c. The different signals were generated under impact conditions, as shown in Fig. 15d. The combined 3D printing technology with self-powered sensors play an essential role for long term space missions, their protection, and restoration of the space station field [119]. Apart from that, several studies have been reported to demonstrate the potential of adaptive surface materials, that can utilize the TENG based actuation and sensing for debris cleanup. For-example, Nie et al. developed the electrically responsive materials/devices based on triboelectric effects for electrostatic gripping [122]. Moreover, several other researchers, have developed the flexible tactile sensors which may be utilized as real-time contact sensors during debris collection operations [123–125]. Hence, the self-powered TENG sensors are essential for monitoring extraterrestrial life, surface features, and safety on alien planets [119]. It will play a crucial role in future space explorations, aiding in the development of human habitats on other planets and providing critical support for scientific research and space missions.

#### Self-Powered TENG Sensors for Deep Space Object Perception

Self-powered sensors play an important role in increasing the sustainability and efficiency of deep space missions and spacecraft. These sensors can harvest energy from various mechanical and physical stimuli, which makes them an ideal candidate for challenging environments without external batteries. Among the different types of sensors, the self-powered triboelectric sensors give a potential path for the harsh environments of space. Self-powered triboelectric sensors are exceptional innovations for the harsh environments of space owing to their efficiency, cost-effectiveness, and sustainability. Researchers have utilized self-powered TENG sensors for space object perception and for monitoring space debris.

**Fig. 16:**
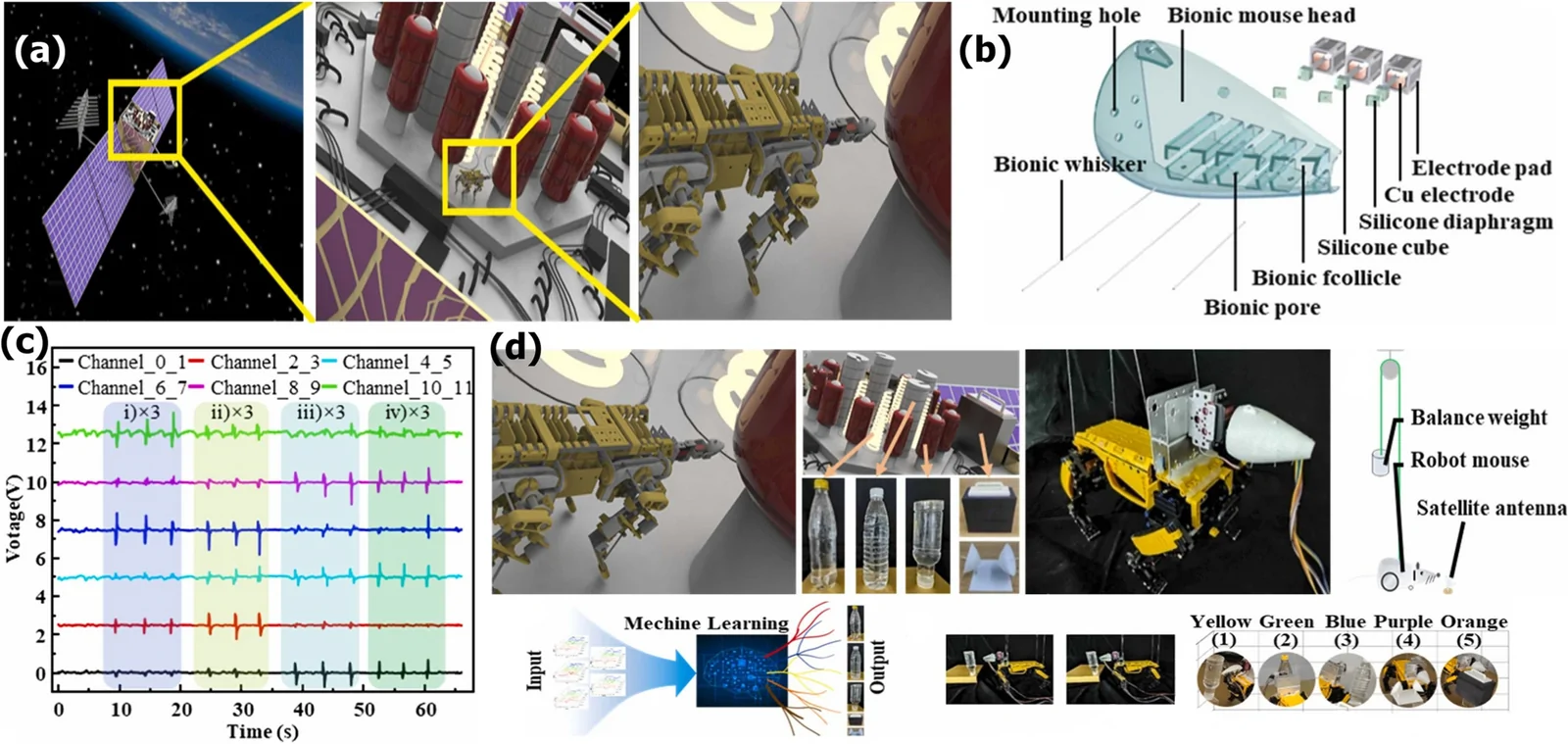
Self-powered TENG sensors for deep space object perception**: a** Schematic of BMWS for satellite surface prediction in dusty environments, **b** structural overview of BMWS, **c** triboelectric output performance of BMWS in different deflections, and **d** Schematic of satellite surface structures, **a–d** reproduced with permission from Elsevier, Copyright@2023 [126]

Space and terrestrial objects perceptions are necessary for the safe landing of spacecraft and to prevent collisions with space debris. A revolutionary advancement has been made in the field of sensors for space object perceptions, but the problems associated with these sensors include cost-effectiveness, sensitivity, and compatibility with harsh environments. Due to TENGs’ advantages of reliability, sensitivity, and compatibility with harsh environmental conditions, self-powered TENG sensors can provide a potential route for space object perceptions [126]. In this regard, in 2023, Hou et al. demonstrated a self-powered, less-weight biomimetic sensor for space object perception [126]. In this work, a bionic self-powered whisker sensor (mouse-inspired) was demonstrated, as shown in Fig. 16b, for the discrimination of the hole width, distance monitoring, identification of the object shape, etc. The self-powered whisker sensors showed a stable and reliable output performance after 6,000 cycles, even at low-temperature conditions (-60 °C). Space traffic handling is necessary in the case of satellite safety because the satellite has a complex natural structure. These satellites are difficult to detect under dark conditions of space. Traditional approaches are unsuccessful under these conditions, and new alternative technologies are required. This proposed self-powered less-weight biomimetic sensor is an effective approach to quietly map unknown satellite surfaces for improved space traffic coordination, as shown in Fig. 16a. Additionally, 1 × 3 whiskers array sensors were fabricated, and obtained signal responses are depicted in Fig. 16c.

This self-powered bionic sensor has the ability to detect the detailed surface shape of satellites in space as demonstrated in Fig. 16d. In addition, Fig. 16d depicts the surrogate shapes that were developed to resemble the actual satellites shapes as well as pictorial image of the Mouse-Bot along with self-powered sensors. Machine learning techniques (artificial neural networks (ANN)) were utilized to differentiate these surface shapes with an average accuracy of 91.0% [126]. This proposed self-powered bionic sensor has also shown excellent capabilities and promising potential for multi-shape recognition [127–129]. Additionally, a recent development in integrating the energy storage and power management modules with TENG sensors demonstrates the practical applications of real-time data transfer and power management [130, 131]. These techniques utilize the charge amplifiers as well as rectifiers and may contribute to the future development of robust, self-powered space systems.

### Comparison of Other Energy Harvesting Technologies with TENGs for Space Applications

TENGs provide a route for energy harvesting as compared to conventional technologies like solar cells, RTGs, and thermoelectric generators in space missions. Each energy harvesting technology has its own advantages and disadvantages. Table 1 shows the pros and cons of each energy-harvesting technology for space missions/crafts. Solar power harvesting is a sustainable, renewable, and stand-alone energy source that has been utilized for long-term space missions. The limitations associated with solar power harvesting include an external battery system and high cost. The efficiency of the solar panels decreases with the increase in the distance from the sun in outer space missions. Nuclear power generation, based on the thermoelectric energy conversion principle, offers a reliable and continuous energy supply for space missions. It is highly efficient, requiring only a small amount of uranium to produce substantial energy. However, the risk of nuclear accidents remains a significant concern, particularly during launches and operations. Additionally, this energy source faces limitations such as security threats and its non-renewable nature, which pose challenges for long-term sustainability in space exploration. In addition, advanced self-powered TENG is an alternative and efficient energy source for space missions as compared to conventional technologies owing to its lightweight, self-powered nature, no safety issues, high output power, flexibility/sustainable nature, and low cost. Hence, from Table 1, it can be concluded that TENG devices provide a potential solution for efficient energy harvesting in challenging space environment conditions. Moreover, Table 2 summarizes the comparison of benefits/limitations of TENG based systems vs alternative technologies for space applications.

**Table 1:** Comparison analysis of pros/cons of the energy harvesting technologies for space application/missions

Power generation system	Type of mission	Advantages	Disadvantages	Energy density	Environmental adaptability
Solar power generation	For Inner planets and long-time missions	Sustainable and renewable energy source Stand-alone power generation	Energy storage system needed, Dependence on day and night cycles and angle of incidence of light on the solar arrays, Expansive energy source, Big Size is required for solar cells	~ 300–500 W m^−2^	Limited by light access; affected by radiation,
Nuclear power generation	For outer planets and long-time missions	Less amount of Uranium is necessary for production of large amount of energy No effect of Environmental Conditions Cost-effectiveness	Security threat Non-renewable Happening of nuclear accidents Large amount is required for safety equipment	~ 60–100 W kg^−1^	Excellent in dark and cold conditions, deep space; required radiation protection
Electromagnetic Energy harvesting	For long-time missions	Light weight Continuous power supply Less cost is required for maintenance	Energy density, Effect of environmental conditions, Not a mature technology	~ 10–100 Mw m^−2^ (high variability)	Not very attractive in deep space
Advanced self-powered Triboelectric nanogenerators/sensors	For all Space Missions and satellites	Lightweight operate under harsh environmental conditions Self-powered nature No safety issues Less maintenance requirements Flexible and sustainable Suitable for low power applications	Energy density Scalability Integrated complexity	~ 10–100 mW m^−2^	Operates in vacuum, microgravity excellently and CO₂-enrich or UV-rich conditions, − 125 °C to + 400 °C

**Table 2:** Comparison analysis of benefits/limitations of alternative technologies vs TENG-based systems for space applications

Application	Target function	Operating conditions	TENG benefits	Limitations vs alternatives
Planetary Exploration missions	Harness the mechanical energy from wind, and dust removal on Mars	Low pressure (600 pa), Low temperature (− 120), and high CO_2_ environment	Lightweight, cost-effective, and operates in low light	Less power vs solar performance
Manned Space equipment	Powering the wearable gadgets integrated with space suits and gloves	Various temperature ranges	Self-powered, flexible and high temperature resilience	Materials durability for long-term usage compared to batteries, Integration complexity
Deep space object perception	Mongering the space debris	Collision and impact monitoring and low temperatures	Quick response, required no external batteries	Less resolution for limited space debris compared to ground radar
In-orbit robotic operations	Robotic Systems for In-Orbit Assembly	Microgravity and wide temperature ranges	Compact, autonomous energy source, terrain sensing	Complex calibration as compared to solar cells and wired sensors
Spacecraft health and structural monitoring	Examine the stress and health of space craft systems	Vacuum, microgravity, vibration	Real time sensing and integration with space structures	Calibration problem compared to piezoelectric devices

Researchers have been attracted to the development of advanced self-powered TENGs for space missions. Table 3 shows the comparison analysis of the performance metrics of self-powered TENGs for the Martian dust environments, spacecraft flywheel health monitoring, space suits, and space extravehicular activities.

**Table 3:** Comparison analysis of the performance metrics TENG and self-powered triboelectric sensors for space application

Applications	Modes of operation	Type of Energy harvested	Electrical Performance	Refs
Triboelectric nanogenerators for Mars Environment	Vertical contact separation	Harvested mechanical Energy through mechanical feedthrough	V = 130 V, I = 7.99 µA	[11]
Self-repairing parachute based on triboelectric collision sensor for Martian dust environment	Contact separation process	–	V = 0.6 V	[87]
Triboelectric Sensor for space application (Phase A design)	Contact separation process	Harvested energy from small motions and vibrations	V = 98 V	[91]
Self-sensing Triboelectric nanogenerators for space craft flywheel health monitoring	A floating freestanding mode	Harvested energy from bearing and load of space craft flywheel	V = 90 V, I = 9 nA P = 79 μW m^−2^	[66]
Hybrid tribo-piezoelectric nanogenerators for the implementation of space suits	Vertical contact-separation	Harvested the energy form human body movements	V = 26.4 V	[65]
Aerogel triboelectric nanogenerators for harsh conditions of space	Vertical contact-separation	Harvested the mechanical energy	V = 135 V, I = 6 µA P = 31.9 mW m^−2^	[97]
Self-attaching robotic triboelectric sensor for in-orbit spacecraft application	contact–separation process	–	V = 4 V	[118]
Robotic bio-paw triboelectric sensors for space surface identification	contact–separation process and sliding mode	–	V = 200 mV	[50]
3D printing based flexible self-powered triboelectric nanogenerators	Single electrode mode	Harvested the ambient mechanical energy	V = 65 V, I = 90 nA	[119]
Multimodal triboelectric tactile sensor for space extravehicular activities	contact–separation process	Harvested the energy form human body movements	V = 11.4 V Accuracy = 95%	[92]
Self-powered mouse whisker sensor for space object perception	contact–separation process	–	V = 0.2 V	[126]

### Role of TENG Devices in Satellite Systems

**Fig. 17:**
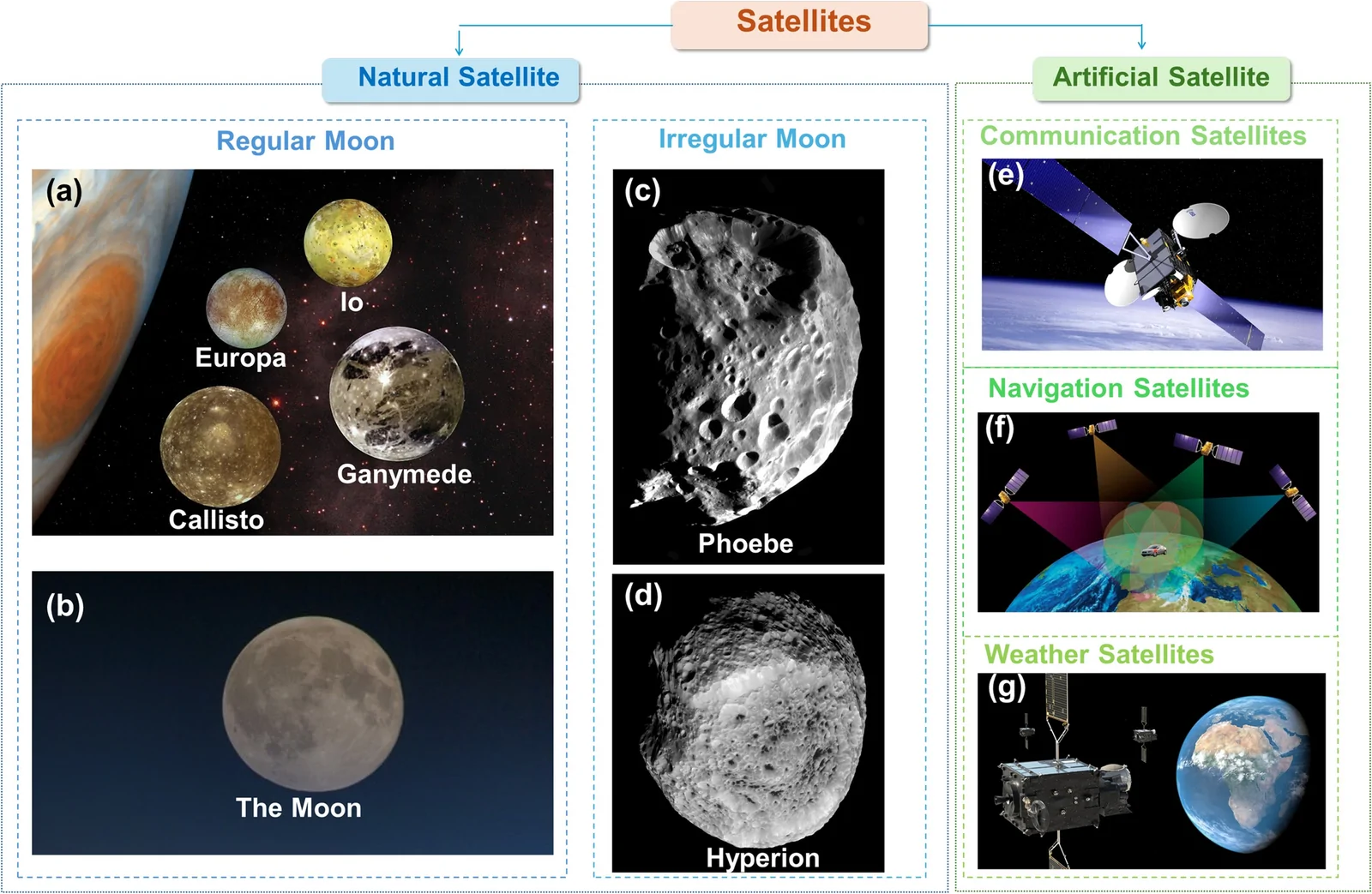
Classification of satellites. **a, b** Regular moons which have nearly circular orbits and prograde motion, **a** the moons of Jupiter: Io, Europa, Ganymede, and Callisto, **b** Earth’s Moon. **c, d** Irregular moons characterized by eccentric, often retrograde orbits: **c** Phoebe, **d** Hyperion. Images credit of (**a–d**): NASA. **e–g** Artificial satellites categorized by functionality: **e** communication satellites, **f** navigation satellites, **g** weather satellites. Images credit of (**e–g**): European Space Agency (ESA)

The artificial objects that can be put into orbit around different planets, especially Earth, to promote a variety of functions like communication and navigation, etc., are known as satellites. They are divided into two major types: natural satellites and artificial satellites, as demonstrated in Fig. 17. The natural satellites are further classified into two types such as regular moon as shown in Fig. 17a, b, and irregular moon as shown in Fig. 17c, d, while artificial satellites are classified into several types such as earth observation satellite, communication satellites (Fig. 17e), navigation satellites (Fig. 17f), and weather satellites as shown in Fig. 17g. Both artificial and natural satellites play an essential role for the better understanding of the space missions. Further, energy harvesting is an important aspect of increasing the efficiency and sustainability of satellite operations. The most common types of energy-harvesting technologies in satellite missions are photovoltaic power systems and nuclear power systems. Limitations of using solar panels include their huge reliance on sunlight that could not working well in the low or no-light areas of Earth, while nuclear power systems could also increase the cost of satellite missions. These drawbacks underscore the necessity for dependable energy-generating options. Hence, self-powered sensors/nanogenerators are gaining momentum in satellite operations due to efficiency, sustainability, cost-effectiveness, and autonomous operations. In this section, we will summarize the developments of self-powered triboelectric nanogenerators/sensors for satellite missions.

**Fig. 18:**
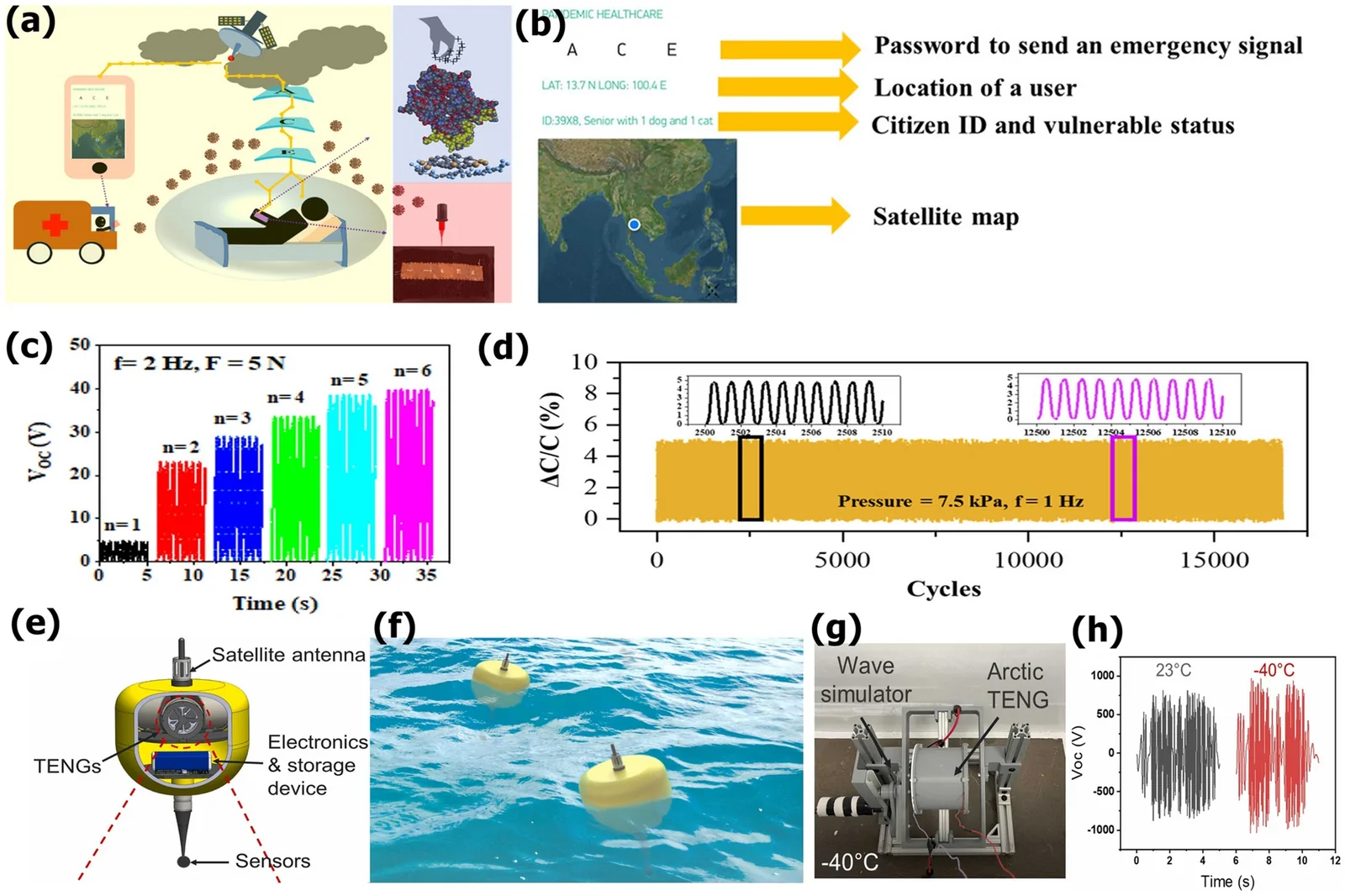
Role of TENG devices in satellite systems** a** Schematic overview of the self-powered triboelectric to initiate the satellite communication system, **b** demonstrated mobile application showing a satellite map above the earth and a password to initiate the satellite signal, **c** output voltage of the SETENG, **d** durability of the electrical performance of the SETENG for 17500 cycles, **a–d** reproduced with permission from American Chemical Society, Copyright@2017 [136]; **e** schematic overview of the Arctic-TENG’s for satellite communication system, **f** design of the free-standing Arctic TENG’s system, **g** testing/characterization setup for Arctic TENG in chest freezer, and **h** electrical performance of the Arctic TENG at room as well as extremely low temperatures, **e–h** reproduced with permission from Elsevier, Copyright@2023 [137]

Satellite communication systems are the new promising technology that transmits information from one location to another through the satellites in orbit around the earth [132]. A communication satellite is a type of artificial satellite that can also transmit signals between different locations on Earth [133]. Advancements in satellite communication systems have successfully incorporated self-powered triboelectric sensors, which employ the TENGs technology to increase their operational abilities. In this regard, Hong et al. developed seesaw structured based self-powered triboelectric–electromagnetic hybrid nanogenerators for sea surface wireless positioning [134]. In this work, authors utilized global positioning system (GPS) module for sending and receiving the information in the smart marine field. Moreover, Zhao et al. underwater wireless communication driven by TENG devices in complex underwater environments [135]. These self-powered triboelectric sensors can produce electrical power independently by harvesting the ambient energy sources without any external batteries. These sensors have attracted great attention toward satellite communication systems due to their capability to operate continuously without any external sources. This technology not only increases the reliability of satellite communication systems but enhances their operation in harsh and challenging environments. The integration of triboelectric sensors in satellite communication systems allows users to send emergency requests for instantaneous help, which improves the efficiency of response efforts [136]. In this regard, in 2021, Kalasin et al. demonstrated a self-powered wearable triboelectric sensor that can send emergency requests quickly through satellite signals [136]. The schematic overview of the satellite communication with a self-powered triboelectric sensor is shown in Fig. 18a. The mobile application showing a satellite map is showing in Fig. 18b. The single-mode triboelectric sensor (SETENG) was integrated with satellite technology, which can transmit the signals through the satellite efficiently. The output performance of the proposed device was investigated, as shown in Fig. 18c. In addition, the mechanical stability of the device was also investigated, as shown in Fig. 18d. Relying on the fast accumulated charges on the user’s finger and the electrets, the renewable, sustainable energy could activate the wearable sensor to communicate with the satellite and locate the user, which would be beneficial for vulnerable populations when needed. Additionally, the TENG device showed a remarkable electrical output performance even at low temperatures (− 20 °C), indicating that TENG is a promising candidate at low-temperature conditions. However, the research on TENG devices for harsh weather conditions (− 40 °C) is lacking. In this regard, Jung et al. reported an Arctic TENG, which produces electrical power to drive satellite communications systems in Arctic conditions [137]. The proposed TENG device was designed for extremely low temperatures as well as cold temperatures as compared to room temperatures [137]. The Arctic TENG generates a power density of 21.4 W m^−3^, which is a sufficient energy requirement to drive the satellite communications systems. The conceptual and schematic of the proposed design as shown in Fig. 18e. In this proposed work, the freestanding TENG mode was used to harvest the energy at its maximum limit from the low-frequency waves of the ocean as shown in Fig. 18f. The electrical performance of the Arctic TENG was investigated at − 40 °C in the chest freezer, as shown in Fig. 18g. The open circuit voltage of the Arctic TENG was measured at 23 and -40 °C as shown in Fig. 18h. The voltage of the voltage of the Arctic TENG was 250 V higher at low temperatures as compared to room temperatures. Despite this analysis, the Arctic TENG showed a remarkably stable performance for over 1 years’ worth of wave cycles. This proposed system can generate energy of 8.59 kJ/year, which is enough for a satellite communication system [137]. Hence, this Arctic TENG showed a promising solution for the long-term working of the satellite communication system.

## Challenges of TENGs for Space Applications

**Fig. 19:**
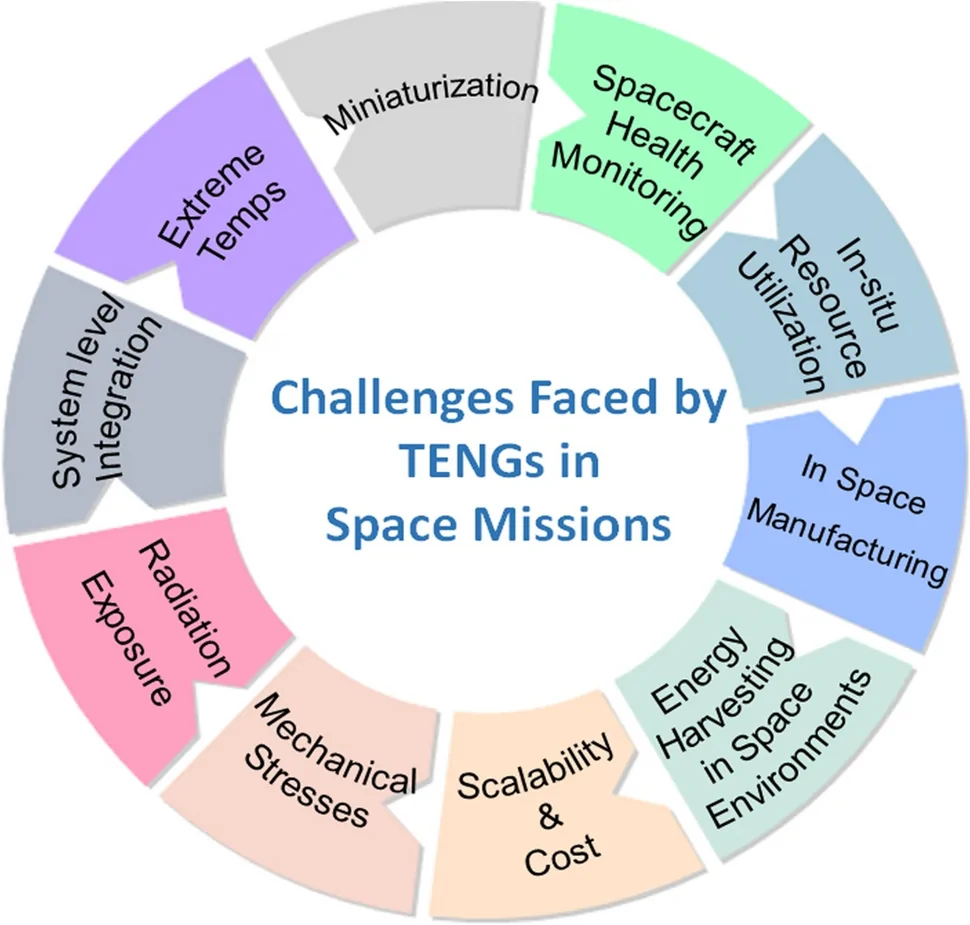
Overview of major challenges faced by TENGs devices in space missions

TENGs have a lot to offer for space missions, yet there is a list of challenges that must be dealt with before making it into practical applications [11, 59]. These challenges include the sensitivity toward extreme temperatures (spanning from 150 °C and above) [138, 139], vulnerability to radiation effects (can exceed 500 millisieverts (mSv) each year) [140–143], nearly vacuum settings (~ 10^−6^ to 10^−9^ Pascal (Pa)) [144–147], durability under high-stress environments (impacts from meteoroids traveling at speeds up to ~ 72 km s^−1^) [148–151], and effective energy management (Fig. 19). Solving such hurdles will determine how well TENGs function and last over time during space missions [152, 153]. The energy harvesting efficiency of TENGs can decrease by more than 40% under hot or cold conditions [154, 155]. TENG devices have a huge potential to become a game changer for space missions by producing energy and monitoring spacecraft conditions that could directly influence the future trajectory of space exploration [156–158]. TENG systems were able to harvest energy from sources such as vibrations and wind, which has attracted interest in its potential use in the field of space technology for powering spacecraft systems [90, 159, 160]. However, TENG devices must overcome challenges related to harsh space conditions to enhance their efficiency. This section addressed the potential challenges faced by TENG technology during space expeditions [161–164]. Addressing these challenges could transform energy generation for spacecraft, benefiting small satellites like Cube-Sats and future planetary expeditions.

### Extreme Temperatures

Extreme space temperatures pose great challenges for TENG devices as they could directly affect the overall efficiency and longevity of these energy-harvesting devices. The temperature ranges on Moon ranges from 127 °C (260 °F) during the day to 173 °C (280 °F) at night; while Mars experiences temperatures ranging from about 20 °C (68 °F) to 125 °C ( 193 °F) [165, 166]. Sudden temperature shifts could cause abrupt expansions and contractions in TENG materials, leading to stress, cracks, and wear over time [96, 167]. The previously tested polymers like polyethylene terephthalate (PET) were not suited for fluctuations as PET degrades at temperatures exceeding 70 °C (158 °F) [168, 169]. More resilient fluoropolymers such as polytetrafluoroethylene (PTFE) or fluorinated ethylene propylene (FEP) could endure temperatures up to 260 °C (500 °F) but might still struggle in extremely cold conditions where their effectiveness is reduced due to increased brittleness, affecting the charge transfer and energy output. Studies indicate that TENG efficiency decreases by 20%-30% with cycling, as prolonged exposure weakens surfaces and disrupts energy conversion consistency over time. To address these challenges, researchers are exploring the use of high-durability polymers that can work efficiently in much higher temperatures up to 400 °C (752 °F) [170, 171]. Nanomaterials like graphene are also been explored for space exploration to improve stability, radiation resistance, and overall durability [172–174]. Self-healing polymers that could repair cracks caused by extreme temperature changes are also important for space missions that could prolong the lifespan of TENG devices.

### Exposure to Radiations

Radiation in space poses a serious challenge to TENG technology as prolonged exposure to energetic particles like protons and electrons can degrade commonly conventional materials, such as polymers and perovskite [175, 176]. This material breakdown (due to harmful radiations) could directly impact the strength and electrical properties of these materials and the overall durability/efficiency of TENG devices. Thus, it is extremely important to emphasize the importance of radiation-resistant materials for extended space missions. Space radiation from sources such as solar particle events (SPE), galactic cosmic rays (GCR), and the Van Allen belts encircling the Earth could impact chemical compositions and result in ~ 30% decrease in energy production at doses near 10 kGy levels [177, 178]. This radiation exposure could give rise to two types of material deterioration, including polymer chain breakage (PCS) and irradiation-induced liquid damage (ILD). To boost the durability of TENG devices in the space environment, it is important to utilize materials that are radiation resistant, which can be achieved by applying protective coatings that can withstand radiation levels as high as 100 krad. The integration of radiation-hardened semiconductor switches and controllers into the design of TENG systems could significantly enhance their performance in environments with high levels of radiation such as space and planetary surfaces. It is essential to test these materials in both simulated and real-life environments to develop TENG devices for space missions and ensure reliable energy generation in harsh conditions.

### Mechanical Stresses and Impact Resistance

TENG devices in space are subjected to significant mechanical stresses due to vibrations (0.01 to 5 g during launch, landing, and typical operations), microgravity effects (0.1 to 1 Hz), shocks (can reach 1000 g or higher), and impacts from micrometeoroids (traveling at speeds of ~ 10 to 40 km s^−1^) and debris (traveling at speeds of 28,000 km h^−1^). These mechanical stresses and impact resistances could compromise the durability and efficiency of TENG devices during space missions. During launches, spacecraft experience mechanical vibrations ranging from low-frequency (10-50 Hz) to high-frequency (over 1000 Hz) oscillations, with g-forces reaching up to 30 g. Such stresses can substantially weaken TENG materials, leading to layer separation and degradation in triboelectric components like polymers and metal electrodes. Additionally, high-speed micrometeoroids, traveling up to 70 km s^−1^, also pose a severe risk by puncturing protective layers, causing delamination, and disrupting the charge transfer process. These impacts are common in low Earth orbit, on the Moon and Mars, where TENG devices must be able to withstand frequent collisions. Extended strain from repetitive contact-separation cycles also leads to mechanical fatigue (reducing elasticity and energy output). Studies showed that PDMS-based TENGs experience a 20% drop in efficiency after 1000 stress cycles. Continuous friction causes surface wear that reduces charge transfer efficiency and leads to unstable power generation. Developing impact-resistant and self-healing materials with real-time health monitoring systems is essential to maintain triboelectric properties and enhance the resilience of TENG devices in space (despite continuous mechanical stress and surface damage). These advancements enable TENG devices to ensure reliable energy production and integration with energy storage systems like supercapacitors or batteries during demanding space missions.

### Scalability and Cost-effective Manufacturing

Ensuring scalability and cost-effective manufacturing of TENG technology is essential to meet the energy harvesting needs of space missions for small platforms like Cube-Sats and large spacecraft. The increasing number of satellite launches (expected by 2030) would develop a need for reliable power sources like TENGs, leading to high demand for its scalable and cos-effective production (from prototypes to mass production). However, challenges lie ahead in maintaining device performance while reducing fabrication complexity and costs during the shift to large-scale manufacturing. Conventional manufacturing methods for TENGs require layering processes that could be expensive and time-consuming when tailored to capture energy from movements or vibrations effectively. Combining polymers with nanomaterials like graphene or CNT could enhance the durability of TENG devices in space to tackle materials science challenges, though it may raise production costs. Thus, further research is necessary to find cost-effective yet long-lasting materials that meet the standards for space applications. 3D printing and Roll-to-roll (RTR) printing techniques could offer a viable manufacturing solution for the efficient mass production of TENG units at lower costs that could substantially cut expenses [179]. Such advancements could be achieved with collaborative efforts among space organizations in conjunction with educational institutions and businesses to secure the dependable integration of TENG technology in upcoming space ventures.

### Unpredictable Energy Generation in Space Environments

The changing space vibrations and solar winds pose obstacles to maintaining a steady energy supply from TENG devices during space missions. The TENG technology could harness energy from spacecraft vibrations as the impact from micrometeoroids and movements of rover wheels; however, the power output tends to fluctuate due to the different levels of intensity generated from these sources. In missions near Earth’s orbit, the regular spacecraft vibrations are advantageous, but that might not be the case in space or planetary missions with lower levels of mechanical inputs. Environments like wind and micrometeoroid impacts add to the unpredictability of energy generation in space using TENG technology over time. Vacuum conditions and extreme temperatures in space, along with low gravity and altered friction, also challenge TENG performance, particularly on surfaces like the Moon and Mars. Dust storms on Mars have hindered equipment like the Opportunity rover's panels (dropped from an average of 600 W-hours per Martian day to as low as 128 W-hours) and could similarly wear down TENG surfaces, reducing contact quality and energy collection. Given that CubeSats and other small satellites function in space under challenging conditions, it is essential to choose materials that can withstand extreme temperatures, radiation exposure, and impacts from debris. TENG energy production in space necessitates advancements in material science and predictive algorithms alongside improved energy storage technologies to guarantee a constant power supply to address these fluctuations across different stages of space missions. Future TENG models must use materials that withstand extreme temperatures and physical stresses to ensure reliable energy generation for ISRU and other space missions during lunar and Martian expeditions.

### Miniaturization for Small Satellites and Cube-Sats

Miniaturization is important for developing energy collection technologies in Cube-Sats, where weight and space constraints limit component sizes. A single unit of Cube-Sats typically has a weight of ~ 1 kg with dimensions of 10 cm × 10 cm × 10 cm with common configurations including 1U, 2U (20 cm), and 3U (30 cm). The importance of miniaturization for small satellites can be deduced from the stat that ~ 1500 CubeSat satellites have been sent into space (since 2023). The cost of building these miniaturized satellites could vary between $ 50,000 and $ 500, 000 based and their operational period is typically around 1 to 3 years in the Earth orbit before their orbits decay. Given these specifications, TENG devices need to be both small in size and capable enough to generate power (~ 5-10 watts) to support essential functions like communication systems and scientific sensors. However, it is a difficult task to decrease the TENG device size while maintaining power output because power generation in these devices is directly proportional to their surface area. The use of advanced materials and innovative designs could only ensure the same energy output even with the small size of TENGs [66, 91, 180]. Flexible materials allow TENG devices to conform to satellite frameworks, saving space and ensuring effective energy capture from vibrations, impacts, and micrometeoroid hits during operation. Stacked TENG designs could enhance power density without increasing device size by using nanostructured materials with high surface area-to-volume ratios. Techniques such as nanofabrication and micro engineering could enable the development of compact TENG configurations that deliver efficient power output. TENG gadgets could play a significant role in CubeSat power setups through progress in material studies design methods and by providing energy solutions that are space-friendly.

### Testing Protocols and In-space Manufacturing

It is mandatory to conduct rigorous testing of TENG devices to confirm their durability in space environment by checking their resistance to vacuum pressure, extreme temperature changes, radiation exposure, and strong mechanical impacts. This validation process involves utilizing vacuum chambers to replicate the conditions of a near vacuum environment and using radiation chambers to imitate exposure to high-energy particles. These tests would ensure that the performance of TENG devices meets the requirements of specific space missions before they are deployed. Moreover, the ability for space manufacturing and repair could substantially improve mission sustainability by decreasing reliance on Earth resources and enabling mission strategies. Despite the challenges of manufacturing and testing in space, emerging technologies like TENGs advance materials science by setting new standards and fostering innovation to make TENG technology an environmentally friendly energy option for intricate space expeditions. These advancements will be an important step forward in ensuring the longevity and triumph of spaceships powered by TENG technology for more intricate space missions. On-site manufacturing of TENG technology could be made possible through fabrication methods like 3D printing through in situ resource utilization (ISRU). This feature would help to decrease dependence on resources from Earth.

### Predictive Maintenance and Spacecraft Health Monitoring

TENG devices could be promising for monitoring spacecraft health by detecting mechanical wear in components like flywheels and bearings. TENG devices could help in predicting maintenance needs and extend the longevity of space missions. Optimal integration of algorithms with data from TENG outputs could allow these devices to oversee integrity levels, accurately detect signs of wear, and predict potential malfunctions for deep space missions where human intervention is limited. TENG systems could offer a two-way advantage by not only generating power but also supporting health monitoring functions. However, challenges persist in ensuring energy production with minimal material wear and enhanced data precision. TENGs rely on vibrations that are abundant during launch but diminish during long space expeditions hence, limiting continuous monitoring power availability. Moreover, TENG materials usually consist of polymers and electrodes that must endure extreme space conditions. Energy data must be translated accurately to provide insights into the wear and structural condition of spacecraft components for its health monitoring using TENG technology. TENG sensors need to be highly sensitive to detect health-related problems of spacecraft (micro-cracks or misalignments) in real time to avoid overlooking essential maintenance requirements. Furthermore, the inclusion of TENG-based monitoring systems into a spacecraft data network should be smooth to ensure integration with onboard infrastructure for data transmission to mission control. Significant advancements in self-sustaining sensors, predictive maintenance algorithms, and robust materials are a way forward to maximize TENG effectiveness in space missions and enhance spacecraft reliability [126, 181–184].

### In situ Resource Utilization and Sustainability

In situ manufacturing (ISM) is becoming important for sustainability (by reducing reliance on Earth resources and enabling part production/repair in space) as we explore the Moon, Mars, and beyond. The development of TENG devices for ISMs is no feat due to a host of challenges it poses along the way. One major challenge is to find materials for TENGs that can seamlessly integrate with ISMs using methods like 3D printing [185–187]. The unique constraints of operating in microgravity environments further complicate matters by presenting challenges like flow control and bonding issues resulting in the solidification of conventional materials. Methods like photo polymerization and sintering techniques are being explored to tackle such challenges. Self-repairing or radiation materials are also being considered to improve the durability of TENGs for longer missions.

One exciting direction, for ISM’s future, would be to utilize resources like regolith or Martian soil to create TENG components that could cut down mission expenses by decreasing the reliance on importing materials from Earth [11, 188]. The lunar regolith is mostly made up of materials like plagioclase and pyroxene (mixed with glass and volcanic debris particles) of sizes ranging from micrometers to a few centimeters in size (~ 1 to 2 mm in diameter) [189–193]. Material like silica and alumina, alongside metal oxides (present on the moon’s surface), could potentially be processed for energy uses. The TENG technology could potentially be used to harness energy on planets like Mars by capturing vibrations and wind energy to aid in on-site resource utilization (ISRU). TENG devices have the capability to convert these mechanical forces into power that could then be utilized for space exploration and resource extraction activities such as mining/extracting oxygen from the environment. However, environmental challenges still exist because the Moon does not have an atmosphere for wind energy utilization, and the thin atmosphere of Mars (with low wind speeds of 10 to 20 m s^−1^) presents challenges in terms of energy generation potential [194]. TENG devices must withstand radiation, extreme temperatures, and material degradation to function reliably during prolonged ISRU operations, such as extracting oxygen or mining ice on Mars. Energy can potentially be harnessed by incorporating TENG technology into tools like drills during these activities to supplement the power needs of the machinery and develop a self-sustaining energy cycle. Organizations like the National Aeronautics and Space Administration (NASA) have developed tools like the Astrobee robot (approximate size and weight of 30 cm and 1.5 kg) for maintenance tasks aboard the International Space Station (ISS) [195]. Such highly sophisticated and advanced tools might also aid in automating TENG repair and assembly procedures in space. Advancements in ISRU and ISM by incorporating TENG technology have the potential to support space missions efficiently and bolster self-reliance while encouraging eco-friendly exploration of celestial objects.

### System Level Integration

TENGs demonstrates a great potential for energy harnessing/sensing in space and extraterrestrial missions, but a major challenge in space missions is their system level integration within spacecraft energy architectures. Successful implementation of TENG devices in space missions required a reliable and independent system comprising power management circuits, energy storage units, and data acquisition systems. The output performance of TENG (pulsed signals and low current) differs significantly form batteries and energy storage modules which is the main key challenge in this area. Power management circuit including rectifiers as well as energy storage suits will be required to address this key challenge. Furthermore, physical integration of TENGs possess thermal and mechanical. Hence, integration of TENGs with spacecraft equipment’s requires engineering approach that addresses a key challenge in this area such as electrical compatibility and mechanical integration. Thus, overcoming these challenges is key to analyze TENGs as powerful components in future space missions.

## Future Directions

**Fig. 20:**
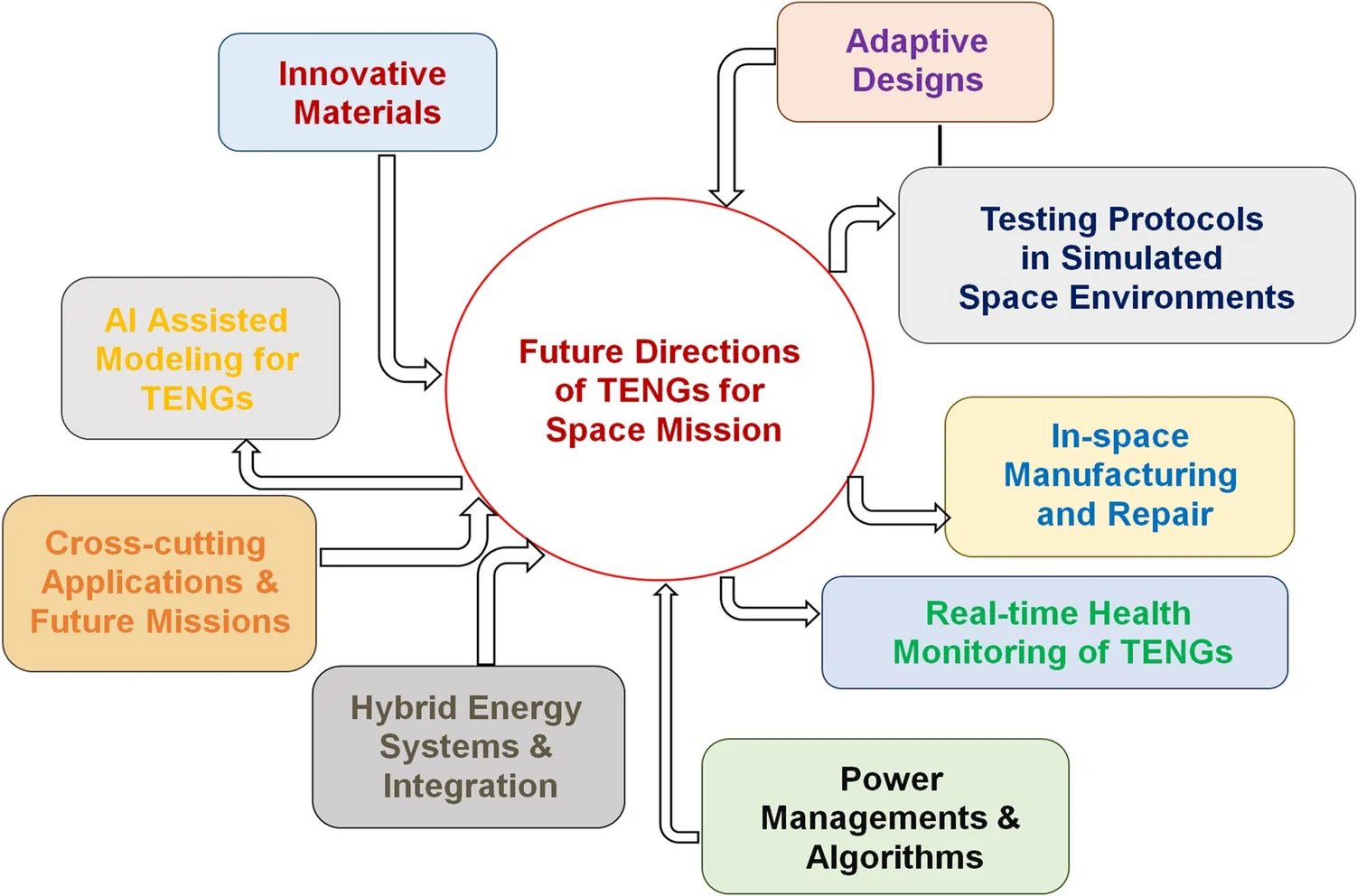
Overview of future developments of TENGs devices in space missions

The next step (future) of the TENG technology for the space/satellite missions includes the innovative materials, adaptive designs, AI-assisted modeling for TENG devices and real-time health monitoring. This sections explains the overview future roadmap of TENG technologies for space environments (Fig. 20). These future directions aim to reduce the current challenges and ensure the robust development of self-powered TENG sensors/harvesters in planetary missions and future deep-space.

### Innovative Materials

The choice of materials for space applications is considerably more stringent than those of earth-based applications. Considerations such as longevity, reliability, harsh environmental resistance, high specific strength, and compatibility with propellant, along with several other factors, make the selection of material a considerably more challenging task than earth-based applications for similar tasks. The current state-of-the-art TENG devices have been developed on some basic triboelectric materials like aluminum. For example, Seol et al. have developed the TENG devices based on common triboelectric materials like aluminum and PTFE. But these materials do not meet all the requirements in space missions. Hence, utilization of advanced materials to fabricate the TENG devices presents a promising route toward the durable energy solutions in space missions. Nonetheless, graphene and other 2D materials are considered an emerging class of materials for space applications [196–198]. This class of materials could play a significant role in advancing TENG technology by enhancing the charge generation efficiency and storage for energy harvesting in space environments [177, 199]. Mxenes are known for their good conductivity and flexibility, which make them well-suited for TENG devices [200–202]. Another category of materials is self-repairing polymers with the ability to autonomously mend damage, ensuring TENG device longevity by restoring properties without frequent maintenance [203, 204]. Self-healing materials can fix themselves to regain 70% to 80% of their original functionality following exposure to radiation or physical strain. Exploring materials that are resistant to radiation (protective coatings) could provide a layer of protection shielding TENG systems from radiation effects [205–207]. Materials such as PTFE and FEP are known for their resilience to temperatures ranging from 190 to 260 °C. These materials could also withstand radiation levels of up to 200 kGy without any significant damage to their structure. Moreover, integrating graphene and carbon nanotubes (CNTs) into these materials has been found to improve their effectiveness by enhancing charge separation and resistance to radiation. Metal-organic frameworks (MOFs) is another material category that can tolerate temperatures up to 400 °C and offer designs to enhance charge generation capabilities [208]. Materials like Kevlar and carbon fiber composites offer higher tensile strength (~ 5000 MPa) and thermal stability (~ 400 °C) thus, enhancing impact resistance. Advancements in nanocomposites, MOFs, fluoropolymers and self-healing technologies could play a massive role in making TENG technology a viable solution for meeting the energy requirements of space missions while promoting sustainability and resilience.

### Adaptive Designs and Hybrid Solutions

Adaptive designs that could offer hybrid solutions are mandatory for TENG technology in space missions. Innovative TENG models need to include ways to resist extreme temperatures in order to work reliably in space conditions. Hence, liquid–solid interface TENGs (L–S TENGs) are the innovative class of TENGs that harness the mechanical energy from liquid motion such as water droplets into electrical form [209–212]. These devices provide an interaction between solid interface and liquid interface to produce the electrical energy. In space habitats, such as lunar bases, or Martian stations where water vapor their fluid movement, and droplet condensation are present, L–S TENGs may serve as self-powered sensors or as innovative energy harvesters. For-example, droplet-based TENG demonstrates an exceptional performance from even single droplet [213]. Thus, incorporation of L–S TENGs spacecraft may provide a new functionality like leak detection, localized power sources for microelectronic devices. Another, successful method could be to add insulating layers like aerogels that are known for their ability to keep heat in to shield the parts from changes in temperature [214, 215]. Improving the design of the TENG structure (with frameworks that evenly spread out pressures) can reduce stress points in areas to enhance durability against frequent vibrations and impacts [216, 217]. A sandwich structure (with layers that absorb energy) could safeguard TENG parts and disperse mechanical energy effectively. Furthermore, utilizing shock-absorbing materials such as elastomers (silicone rubber or fluoroelastomers) or foams can lessen the impact of mechanical forces during takeoff or encounters with debris, thus improving TENG performance [218, 219]. Adding metal barriers (like titanium or aluminum) to TENG designs could protect them against impacts from micrometeoroids and space debris (by absorbing and dispersing the force) [220, 221]. Additionally, incorporating whipple shields (often utilized in spacecraft) could further improve the safety measures for TENG devices [222]. These shields are made of layers that could break upon impact, followed by layers that have the ability to absorb the shocks caused by high-speed debris (moving at speeds over 10 km s^−1^) [223]. Incorporating whipple shields would significantly enhance the resilience of TENG devices during space missions. Moreover, morphing materials such as shape-memory alloys (SMAs) or electroactive polymers (EAPs) embedded with TENGs can create adaptive components, such as deployable antennae, solar panels, or modular habitats [224]. These structures can morph to optimize their performance in response to environmental conditions, such as solar intensity or orbital position while generating power from mechanical deformations during deployment or environmental interactions. Additionally, in planetary exploration missions, surface-deployed sensors must operate autonomously over extended periods [225]. Morphing materials embedded with TENGs can act as self-powered adaptive systems, altering their shape to protect sensitive equipment from extreme temperatures or dust storms while generating energy from environmental interactions like wind or surface vibrations.

### Testing Protocols in Simulated Space Environments

Developing testing procedures specifically for TENG gadgets is important to guarantee their reliability and quality assurance during space expeditions. Entities like NASA and the European Space Agency (ESA) give a lot of importance to these procedures for boosting confidence in the effectiveness of technologies amidst space conditions [226–228]. It is important to conduct testing procedures for TENG technologies in simulated space settings to guarantee their effectiveness and performance during real-time space missions [66]. Tough assessments of TENG devices must be done in vacuum chambers that can mimic the temperatures and vacuum conditions of outer space (with testing temperatures spanning from -150 to + 150 °C) to aid in the identification of material vulnerabilities. Recent studies have been reported that TENG devices have been tested in MARS analog chambers simulating the various temperatures and CO_2_ atmosphere.

Furthermore, to ensure the accuracy and reliability of TENGs in testing for space exploration, advancements are needed to replicate the vibrations and rapid impacts of space travel. Experienced facilities with tools like vibration tables and shock chambers can recreate the launch environment, whereas impact assessment evaluates the efficiency of shielding materials against tiny meteoroids and space debris [229]. Regular monitoring throughout these assessments could offer valuable insights into TENG functionality under real-world pressures. Before using TENG devices on space missions, it is important to conduct lab tests for validation purposes first. It is important that these tests include and simulate the conditions of vacuum, radiation, extreme temperature, and random mechanical impact challenges experienced in space [230]. CubeSat missions enable experimentation in space, thus allowing scientists to assess TENG integration with solar technology/TEGs and gather valuable performance data in space environments [231–234]. Exposure to radiation is a factor that impacts the lifespan of TENG devices in space environments like LEO, geostationary orbit (GEO), and deep space regions. Testing protocols also need to replicate radiation effects by subjecting TENG materials to gamma or X-ray sources for assessing their durability. Furthermore, TENGs need to be able to withstand damage caused by debris and micrometeoroids that requires conducting tests of vibrations and impacts experienced during launch. The materials need to be able to withstand forces to six times Earth’s gravity without breaking down. In space's vacuum, challenges like outgassing and surface wear can impact TENG device performance. It is important to develop materials that can withstand low-pressure environments and also have volatility to ensure that they can generate surface charge effectively. Extensive testing of these materials in simulated conditions is important for developing TENG technologies for space missions.

### Cross-Cutting Applications and Future Missions

TENG devices are seen as a solution due to their ability to provide durable energy that can power communication systems and monitoring devices on spacecraft in a portable manner when solar power may not suffice sufficiently [9, 235]. Advances in TENG technology are important for their durability in space conditions as we look toward the Artemis lunar missions and eventual explorations of Mars. Incorporating TENG devices in vehicles used for space exploration like rovers and habitats on the Moon could provide a supplementary energy solution for prolonged missions [91]. Using in-situ resources (ISRU) can boost TENG energy collection from events like dust storms and seismic activity. This method has the potential to enhance resource extraction by producing electricity from space conditions, showing that TENG technology is a prospective environmentally friendly energy option for upcoming space expeditions.

### In-Space Manufacturing and Repair

In the evolving realm of space exploration (with extended missions on the horizon) it is important to have the capability to craft spacecraft parts while in space to ensure operations. Manufacturing and repairing TENG technology in space offer prospects for advancing space exploration efforts. NASA and other leading space organizations like ESA are exploring the use of automated 3D printing technology aboard the International Space Station (ISS) to develop specific tools and components while in orbit. For-example, in 2023, sun et al., developed a fully 3D printed TENG devices for collision monitoring on space stations. This innovative approach provides the feasibility of in-space additive manufacturing of TENG devices for safety and maintenance monitoring. In-space fabrication allows real-time adjustments, enhancing TENG durability and versatility for space missions. The materials used in TENG gadgets need to be tailored for enduring the demanding conditions of space travel to thrive in success. Harnessing materials for production could notably cut expenses and be useful in critical times. They ought to be robust yet flexible, for methods such as 3D printing. In missions, to space automated manufacturing systems will be key [236–239].

Robotic platforms that can create and fix TENG devices on their own will be essential, for managing manufacturing in zero gravity. This automation will improve the sustainability and efficiency of extended space missions. Future studies ought to center on exploring the practicality of establishing TENG powered monitoring systems for healthcare purposes in space. Through the utilization of printing methods spacecraft could manufacture TENG devices. Promoting ISRU sustainability by producing or repairing TENG components with local Moon or Mars materials will certainly enhance mission sustainability, particularly where resupply is scarce or costly. Using manufacturing to craft TENG gadgets or spare parts from materials discovered in real-time locations in space can be an option going forward. In space expeditions, these advanced manufacturing methods and ISRU capabilities can repair or upgrade worn TENG devices without needing supplies from Earth. This approach (in-space manufacturing and repair) encourages the development of self-sustaining communities to produce items and tools on their own during space travel to improve longevity and environmental friendliness [240, 241]. It is necessary to create testing procedures and improve space manufacturing abilities to effectively incorporate TENG technologies into upcoming space missions that will boost exploration initiatives and prolong mission duration.

### Hybrid Energy Systems and Integration with Existing Technology

Integrating TENGs with TEGs and solar panels could be a viable solution to energy generation challenge during space missions by harnessing multiple energy sources. This hybrid energy generation setup will enable energy harvesting during darkness and convert temperature variations (130 to -270 °C) into electricity to provide power output continuously at a lower cost. Evolving TENG technology can enhance mission longevity, potentially improving spacecraft efficiency for unpredictable conditions on the Moon and Mars. Efficient power management systems (utilizing supercapacitors or batteries) along with hybrid energy setups and AI algorithms are vital for meeting operational needs, especially during low or no light (during eclipses). Additionally, combining TENGs with nuclear energy for ISRU on Mars allows energy capture during dust storms, enhancing reliability. Overall, improving TENG performance through integration with TEGs and solar panels is essential for a sustainable energy framework in upcoming expeditions [242–245].

### Power Management Algorithms

An efficient power management system can play an important role in optimizing the efficiency of TENGs in space missions where energy generation and storage are key priorities [246–249]. Among other challenges of energy, apart from integrating TENGs with other energy-generating devices (solar panels and TEGs), integrating TENGs with energy storage devices (supercapacitors and batteries) is also a challenging task due to their power characteristics. Supercapacitors have lower energy capacity compared to batteries and to address these challenges effectively for ensuring a continuous power supply for spacecraft operations under varying conditions require advancements in Energy Management Systems (EMS). These systems can be made smart by leveraging AI algorithms to balance energy demand and supply while adapting to different scenarios. By merging with machine learning, TENG data could aid in recognizing signs of deterioration like vibrations signaling wear and fatigue triggering maintenance steps like adjustments or fixes, in setup. A spacecraft with an AI-driven smart EMS will be able to manage energy from TENG devices, solar panels, and TEGs under challenging conditions of extreme temperatures and radiation exposure [250]. Batteries have a higher storage capacity but require frequent recharging and contrastingly supercapacitors can withstand over a million cycles. A smart EMS will prioritize tasks during peak demands by turning off critical systems to save energy. Development of an AI-driven smart EMS will switch between TENGs, TEGs, and solar panels while also storing excess energy to be used later in critical situations of upcoming space expeditions.

### Real-Time Health Monitoring of TENG Systems

Monitoring the device condition of TENGs in time will guarantee their durability and effectiveness during space missions. Recently, TENGs devices have been utilized in treadmills of astronauts and flywheel systems of space crafts to examine the stress, motion, and micro-vibrations in real-time. These demonstrations have provided a promising investigations in lab-scale simulations, paving the way for future missions. Additionally, a variety of techniques can be used to achieve this goal including the resonant sonic holography method (RSHM) and embedded sensors within the TENG device structure (to constantly track strain, material degradation and fatigue levels) [251, 252]. Operators will be able to optimize TENG devices, manage risks, and timely maintenance actions by analyzing gathered information to prevent malfunctions. Space exploration missions are growing in complexity and duration that makes it even more important to monitor energy systems in time. Cutting-edge and smart EMS also have the task of assessing the state of TENG devices to spot indications of wear and damage caused by various factors (physical impacts, radiation exposure, and mechanical strain). Incorporating self-sensing features into TENG devices can promptly identify any performance-related issues thus, enabling mission operators to address them and prevent critical breakdowns. Incorporating TENG information into maintenance algorithms can boost effectiveness and dependability in fields like spacecraft technology. Proactive maintenance employs up-to-the-minute data to avert malfunctions and operational hitches. A groundbreaking advancement in this area involves the invention of self-sustaining TENG sensors that are suitable for use on spacecraft missions. These sensors are designed to measure parameters like vibration intensity and temperature changes in spacecraft batteries. This feature proves advantageous during tasks that involve engine operations or component adjustments. TENG-powered sensors have the capability to oversee parts like flywheels, bearings, and vulnerable panels to wear and malfunctions. Transmitting this information to the control center will allow the concerned team to assess the spacecraft's status and anticipate issues, enabling quicker responses to minor vibrations or irregularities. Moreover, TENG gadgets can be combined with health monitoring systems to evaluate the strength of spaceship hulls and frameworks. These self-powered sensors can offer insights into the strains experienced by spaceship structures by identifying cracks or changes in shape caused by impacts. This function is vital for missions encountering space debris, temperature variations, and intense radiation.

### AI-Assisted Modeling and Digital Twins for TENG Deployment in Space Conditions

The innovative technologies such as artificial intelligence (AI) and digital twin (DT) are the transformative growth, showing a new opportunity to enhance the efficiency of TENG systems under space environments. TENG devices are inherently sensitive to environmental variations and materials properties, these computational tools provide powerful avenues for optimization and real-time system adaptation. Apart from that, concept of DT technology, was initiated by NASA, to enhance the reliability and efficiency of space missions. DT technology has been widely used and practically implemented in various aerospace sectors as well as in the field of energy harvesters. For-example, DT technology integrated with AI can monitor the degradation analysis of TENGs under repeated mechanical cycling in Martian or lunar environments, enabling preemptive design updates or autonomous reconfiguration of energy harvesting strategies. Moreover, the AI technologies, machine learning algorithms along with finite element modeling (FEM) can predict the charge accumulation and dielectric breakdown in harsh space conditions. Hence, the integration of AI and digital twin technologies provides a paradigm shift from static to adaptive energy harnessing systems. In future space missions, the integration of AI and digital twin technologies will be important for providing resilient, self-healing, and intelligent TENG-based devices ensuring long-duration, self-powered operation in dynamic and unpredictable extraterrestrial environments.

## Conclusion

This review further builds on progress being made in the advancement of energy harvesting and self-powered sensing technologies, including (TENGs) for space missions and satellite systems. The main goal of this comprehensive review paper is to identify and develop sustainable, efficient, and reliable energy systems that can function in rigid space conditions. Current research focuses on deployment of TENGs as energy harvesters and self-powered triboelectric sensors for space applications. This review also discusses the progress made in conventional sources of energy, for instance, solar panels and RTGs, and their bottlenecks. The first section details the problems with conventional energy systems and presents self-powered nanogenerators and sensors, as well as how TENGs work and the choice of suitable materials. Section 2 reviews the prospect of conventional energy technologies and the new applications of TENGs for planetary exploration missions (Mars environment monitoring), manned space equipment, In-orbit operations/mission management, spacecraft's design/structural health monitoring and aeronautical systems. Section 2.4 discusses satellite systems and how self-powered sensors can be used in satellite operations. The challenges and opportunities, along with future directions and innovations for self-powered TENGs in space missions and satellites, are discussed in Sects. 3 and 4. The key findings of this review papers as given as follows:

1) TENGs provides a viable route to resolve the critical limitations of the conventional energy technologies such as solar cells and nuclear power. Their compact design, lightweight and ability to harness the energy form the mechanical movements make them promising candidate for space applications.

TENGs can be easily integrated with various space systems including space suits, space gloves and spacecraft health monitoring. Their integration into space satellite communications systems could increase the efficiency and sustainability of space missions and can provide a self-sustaining power source.

Self-powered triboelectric sensors can function under extreme environments which makes them ideal candidate for durable space missions.

Thus, integrating self-powered TENGs into space/satellite systems presents a good way of improving power efficiency, sustainability, and reliability for future long-duration space missions in extreme environments. The potential exists for TENG devices is to improve space missions because they provide sustainable, self-powered, and reliable energy solutions that will increase both spacecraft efficiency and duration in harsh environments. Continued research in this key area will overcome the existing challenges and unlock the potential of TENG is space missions.
